# The *Rpv3-3* Haplotype and Stilbenoid Induction Mediate Downy Mildew Resistance in a Grapevine Interspecific Population

**DOI:** 10.3389/fpls.2019.00234

**Published:** 2019-03-06

**Authors:** Silvia Vezzulli, Giulia Malacarne, Domenico Masuero, Antonella Vecchione, Chiara Dolzani, Vadim Goremykin, Zeraye Haile Mehari, Elisa Banchi, Riccardo Velasco, Marco Stefanini, Urska Vrhovsek, Luca Zulini, Pietro Franceschi, Claudio Moser

**Affiliations:** ^1^Research and Innovation Centre, Fondazione Edmund Mach, San Michele all'Adige, Italy; ^2^Ethiopian Institute of Agricultural Research, Addis Ababa, Ethiopia; ^3^Department of Life Sciences, University of Trieste, Trieste, Italy; ^4^CREA Research Centre for Viticulture and Enology, Conegliano, Italy

**Keywords:** disease symptom phenotyping, “Merzling” *Plasmopara viticola*, peroxidase, polyphenols, QTL analysis, stilbenes, *Vitis* spp.

## Abstract

The development of new resistant varieties to the oomycete *Plasmopara viticola* (Berk.& Curt) is a promising way to combat downy mildew (DM), one of the major diseases threatening the cultivated grapevine (*Vitis vinifera* L.). Taking advantage of a segregating population derived from “Merzling” (a mid-resistant hybrid) and “Teroldego” (a susceptible landrace), 136 F1 individuals were characterized by combining genetic, phenotypic, and gene expression data to elucidate the genetic basis of DM resistance and polyphenol biosynthesis upon *P. viticola* infection. An improved consensus linkage map was obtained by scoring 192 microsatellite markers. The progeny were screened for DM resistance and production of 42 polyphenols. QTL mapping showed that DM resistance is associated with the herein named *Rpv3-3* specific haplotype and it identified 46 novel metabolic QTLs linked to 30 phenolics-related parameters. A list of the 95 most relevant candidate genes was generated by specifically exploring the stilbenoid-associated QTLs. Expression analysis of 11 genes in *Rpv3-3*^+/−^ genotypes displaying disparity in DM resistance level and stilbenoid accumulation revealed significant new candidates for the genetic control of stilbenoid biosynthesis and oligomerization. These overall findings emphasized that DM resistance is likely mediated by the major *Rpv3-3* haplotype and stilbenoid induction.

## Introduction

Around 68,000 tons of fungicides per year are used in Europe to manage grape diseases, i.e., 65% of all fungicides used in agriculture although viticulture encompasses only 4% of the EU arable land (Eurostat, 2007). A useful strategy to reduce the impact of pesticides on humans, animals and environment is based on genetic improvement, in particular on the introgression of resistance traits from ancestral species into domesticated varieties. The cultivated grapevine (*Vitis vinifera* L.) is highly susceptible to downy mildew (DM), caused by the biotrophic oomycete *Plasmopara viticola* (Berk. & M. A. Curtis) Berl. & De Toni, the major disease of temperate-humid climate among various pathogen threats.

A total of 27 Quantitative Trait Loci (QTLs) associated with DM resistance in different genetic backgrounds are known and described (VIVC, 2018). In particular, the major *Rpv* loci originated from *Muscadinia rotundifolia* (Merdinoglu et al., 2003), *Vitis riparia* (Marguerit et al., 2009; Moreira et al., 2011), *V. amurensis* (Blasi et al., 2011; Schwander et al., 2012; Venuti et al., 2013), *V. cinerea* (Ochssner et al., 2016), and *V. rupestris* (Divilov et al., 2018). To close the list, the *Rpv3* locus is a major determinant of grapevine DM resistance. Seven conserved *Rpv3* haplotypes were identified in five descent groups of resistant varieties and traced back to their founders, which belong to *V. rupestris, V. lincecumii, V. riparia*, and *V. labrusca* (Di Gaspero et al., 2012). Until now only two haplotypes at this locus were validated in segregating populations derived from different DM resistance donors (Welter et al., 2007; Zyprian et al., 2016).

The related resistance mechanisms are partially known and are due to gene-for-gene recognition, thus signal cascade and finally defense response. A widespread hot spot of NBS-LRR genes was identified within the genomic region where the *Rpv3* locus resides, providing a distinctive advantage for the adaptation of native North American grapevines to resist to *P. viticola* (Moroldo et al., 2008). The defense response also involves the synthesis of secondary metabolites including the stilbenoids. In fact, besides necrosis (e.g., Boso Alonso and Kassemeyer, 2008; Peressotti et al., 2010) and callose deposition (e.g., Gindro et al., 2003), DM resistance can be accompanied by stilbene accumulation (e.g., Alonso-Villaverde et al., 2011; Malacarne et al., 2011; Mattivi et al., 2011) as activated defense mechanisms. To date no study has investigated the genetic basis of polyphenol, in particular stilbenoid, synthesis or its variation upon *P. viticola* infection. QTLs associated with synthesis of most polyphenols are not known; only a few research studies on proanthocyanidins, anthocyanins, and flavonol berry composition have recently been attempted in grapevine (Fournier-Level et al., 2009, 2011; Huang et al., 2012, 2013, 2014; Viana et al., 2013; Ban et al., 2014; Azuma et al., 2015; Costantini et al., 2015; Guo et al., 2015; Malacarne et al., 2015).

In this work, we aim to characterize the locus conferring resistance against *P. viticola* and to identify new polyphenol-related loci in order to shed light on the DM resistance mechanisms in an interspecific segregating population derived from the source “Merzling.'

## Materials and Methods

### Segregating Population

An interspecific segregating population of 136 putative full-sib individuals derived from the cross between the complex *Vitis* hybrid “Merzling,” descending from *V. vinifera, V. rupestris*, and *V. lincecumii*, and the *V. vinifera* cv “Teroldego” was studied. This cross between “Merzling,” mid-resistant to DM and high-stilbenoid producer, and “Teroldego,” susceptible to DM and low-stilbenoid producer, was performed at FEM (Edmund Mach Foundation, San Michele all'Adige, Italy) in 1989. During the 2012 growing season the progeny were propagated as grafted plants in 1L-pots filled with soil:sand:peat:vermiculite (3:1:3:3, v/v) in a greenhouse at 25/20°C day/night temperature, with a 16 h photoperiod and relative humidity (RH) of 70 ± 10%. The propagation of the two parental lines was carried out during 2013.

### Phenotyping

#### DM Resistance Assessment

To collect sufficient fresh inoculum, *P. viticola* propagation was performed on leaves of the susceptible *V. vinifera* cv “Pinot gris” following the protocol by Vezzulli et al. (2018). Six potted plants (PP) per each progeny individual were maintained in two different growth chambers as three biological replicates for *P. viticola*- and mock-inoculation, respectively. Firstly, in June 2012 fully expanded leaves of the 10 week-old PP were *P. viticola*-inoculated (PI) by spraying a suspension of 1 × 10^5^ sporangia/ml onto the abaxial leaf surface and were kept overnight in the dark in a growth chamber at 24°C with 80% RH; mock-inoculated (MI) samples were obtained by analogously spraying distilled water on PP kept under equal conditions. Secondly, in August 2012 the 4–5th leaves from the MI-PP apex of each progeny individual were collected to generate leaf disks (LD) that were inoculated according to Vezzulli et al. (2018). In June and August 2013 the same experiments were respectively repeated on PP and LD of 11 representative progeny individuals along with “Merzling” and “Teroldego” parental lines. Finally, the DM response was evaluated at 8 days post-inoculation (dpi) on PP and at 4, 5, and 6 dpi on LD by means of three parameters evaluated by visual inspection: disease severity (percentage of the disc area showing symptoms of sporulation) and disease incidence (number of discs with sporulation/total number of discs), according to OEPP/EPPO (2001), along with the descriptors OIV 452 for PP or OIV452-1 for LD (overall degree of resistance), recommended by the Organization Internationale de la Vigne et du Vin (OIV, 2009) and adapted according to Bellin et al. (2009) (Table S1A). The latter descriptors, ranking from 1 to 9, are positively correlated with the magnitude of plant response and inversely correlated with the severity of DM symptoms. All percentage data were Arcsin transformed in view of following statistical analysis.

#### Polyphenol Content Measurement

The 2nd−3rd leaves from the PI- and MI-PP apex were collected at 6 dpi and analyzed for the content of 42 phenolics (18 different stilbenoids) by targeted metabolomics (Vrhovsek et al., 2012), according to Chitarrini et al. (2017) with some modifications. For each metabolite, missing values (indicating concentrations below the quantification limit) were imputed by random sampling from a uniform distribution taking values in the range from 0 to the metabolite specific detection limit. Genotype specific levels were estimated as the median value of each metabolite across three biological replicates. To compensate for the expected non-normal distribution of metabolite concentration, genotype-specific data were subjected to logarithmic transformation (ln). Metabolomic data were used to calculate 22 sum/ratio parameters (Table S1B). In all analyses, the difference (delta) between the PI and MI genotype specific values were considered.

### Genotyping and Map Construction

Genomic DNA from the overall 138 studied genotypes was isolated from young leaves using DNeasy Plant Mini Kit (Qiagen, The Netherlands). The 136 putative full-sib individuals and the two parental lines were characterized at the genotypic level by means of 192 microsatellite (Simple Sequence Repeat, SSR) markers, corresponding to an average of ca. 10 SSRs well-scattered along each of the 19 grapevine chromosomes. The microsatellites were chosen based on their polymorphism information in “Merzling” and/or “Teroldego” reported by Salmaso et al. (2008), as well as their unique position and physical distance along the reference genome (http://www.genoscope.cns.fr) (Table S2). The applied mid-throughput genotyping strategy was as reported in Peressotti et al. (2015). Prior to building the “Merzling” × “Teroldego” (M×T) genetic map, SSR markers were tested against the expected segregation ratio using a χ2 goodness-of-fit implemented in JoinMap v.4.1 (JM, Van Ooijen, 2006). Highly distorted (*p* > 0.05) markers were discarded, while the others (*p* ≤ 0.05) were used for linkage analysis unless they affected the order of neighboring loci. Molecular markers were grouped and ordered along linkage groups (LGs) using the Kosambi mapping function implemented in JM. Mapping parameters were set at a logarithm of odds (LOD) value of 8 and at a recombination frequency of 0.45. LG number was assigned according to Adam-Blondon et al. (2004) and the linkage map was visually represented with MapChart v.2.2 (Voorrips, 2002).

### QTL Analysis and Candidate Gene Selection

Genetic map data were integrated with phenotypic data and QTL mapping was performed separately per each experiment by using the simple Interval Mapping algorithm in MapQTL v.6.0 (Van Ooijen, 2009). QTLs were declared significant if the maximum LOD exceeded the LG-specific LOD threshold (calculated using 1,000 permutations) and mean error rate was <0.05. Linkage groups and QTLs were visualized with MapChart2.2 (Voorrips, 2002) and a R *ad-hoc* script (R Core Team, 2018). Through the associated markers, each reliable QTL interval was further anchored and aligned on the assembled version of the grapevine reference genome (Jaillon et al., 2007). For the ease of calculation, base pairs (bp) were converted into centiMorgans (cM) by dividing by 433,989, which is the mean physical distance corresponding to 1 cM derived in this mapping work.

In order to characterize these genomic regions, the 12X PN40024 reference genome (http://genomes.cribi.unipd.it) was exploited to extract version 2 (V2) of the gene predictions (GPs) underlying QTLs. The gene annotation adopted was the one reported by Vitulo et al. (2014) and candidate genes (CGs) were selected adopting the following criteria:

- Proximity to LOD peak offset (in case of large genomic intervals);
- Involvement in trait regulation based on literature (reference genes);
- Assignment to significantly over-represented functional categories. In particular, a Fisher's exact test was applied to evaluate the over-representation of specific functional categories within QTLs controlling a given parameter. Reference to our analysis was the distribution in the same categories of 31,922 12Xv2 GPs assigned to chromosomes (Vitulo et al., 2014). We considered GPs annotated at the third level, with the exception of GPs annotated at the first and second level if not characterized at a deeper level and significantly represented in the genome. Adjusted *p*-values by the Benjamini and Hochberg (1995) method for multiple-testing correction were considered significant when ≤ 0.05. For the functional categories significantly enriched a fold enrichment (Fold-change), as the ratio between the frequency of a category in the QTLs controlling a given parameter vs. the one in the genome, was calculated. The functional categories that passed the Fisher's test and having a Fold-change > 0 were considered as over-represented;
- Involvement in functional categories of interest.

### Quantitative RT-PCR Expression Analysis

Total RNA was isolated from 60 to 80 mg of ground leaves, collected at 6 dpi from each biological replicate (the same powder used for biochemical analysis stored at −80°C), using Spectrum Plant Total RNA Kit (*Sigma-Aldrich*) according to manufacturer's instructions. cDNAs were synthetized using the SuperscriptVILO™ cDNA Synthesis Kit from 1.5 μg of DNAseI-treated RNA (*Thermo Fisher Scientific*). Primer sequences were derived from literature or designed on the reference CG sequence using Primer3 v.4.0 software (http://bioinfo.ut.ee/primer3-0.4.0/) according to MIQE guidelines suggestions (Bustin et al., 2009) (Table S3). Due to the high sequence homology among different family members, it was not always possible to design gene-specific primers. Each primer pair was tested by semi-quantitative RT-PCR on cDNAs from one F1 individual, “Merzling” and “Teroldego” parental lines. The corresponding amplicons were checked by electrophoresis and, prior purification by ExoSAP (*Euroclone*), were Sanger sequenced to verify the specificity of the primer pair. Forward and reverse reads were aligned to the reference CG sequences using Staden Package software (http://staden.sourceforge.net/) in order to confirm their uniqueness.

Finally, the primer pairs that passed this step were employed in qRT-PCR analyses, carried out using the Platinum SYBR Green qPCR SuperMix-UDG in a ViiA™7 thermocycler (*Thermo Fisher Scientific*). The 384-well plates were set up according to the sample maximization strategy proposed in Hellemans et al. (2007). Each sample was examined in three technical replicates, and dissociation curves were analyzed to verify the specificity of each amplification reaction. Reaction conditions and the analysis protocol were the same as adopted in Malacarne et al. (2015). Six housekeeping genes (*VvACT, VvATP16, VvEF1*α, *VvGAPDH, VvSAND*, and *VvUBIQ*; Table S3) were tested for their stability using GeNorm software (Vandesompele et al., 2002). Normalized relative quantities (NRQs) were then calculated by dividing the RQ by a normalization factor, based on the expression of the two most stable reference genes (*VvATP16* and *VvGAPDH*) (Reid et al., 2006).

### Statistical Analysis

Statistical analyses applied to phenotypic data were performed in R (R Core Team, 2018) equipped with tidyverse (Wickham, 2017) https://CRAN.R-project.org/package=tidyverse and ggplot2 (Wickham, 2016) packages. The reproducibility between years was assessed by checking (*t*-test, *p* ≤ 0.05) for the presence of a significant difference in the values of the three phenotypic parameters in a selected group of genotypes. In the case of the 64 polyphenol-related parameters, the reproducibility between years was assessed by testing the difference between PI and MI with a non-parametric Wilcoxon test (*p* ≤ 0.05, corrected for multiplicity applying a Bonferroni correction). The same test was applied to evaluate the presence of a significant induction of the 64 parameters in the PI *vs*. MI samples.

### Phylogenetic Analysis

A total of 90 GPs, including isoforms, encoding peroxidases (IPR000823) were identified in the 12Xv2 PN40024 reference genome (http://genomes.cribi.unipd.it/DATA/V2; Table S6), while the 76 peroxidases from Arabidopsis (Tognolli et al., 2002) were downloaded from NCBI (https://www.ncbi.nlm.nih.gov/). Codon-based alignments of all the coding gene sequences were made using Macse program (Ranwez et al., 2011). Automatically produced alignments of individual genes were loaded into the Seaview alignment editor and manually edited in amino acid mode to discard obviously misaligned regions. The final selection of alignment columns was saved to produce a 206 pos. long curated alignment containing 167 aminoacid peroxidase sequences. Phylogenetic analyses were performed with the help of Iq-Tree multicore version 1.6. beta4 (Nguyen et al., 2015). All sequences in alignment passed χ2 test of compositional heterogeneity (*p* < 0.05). The optimal substitution model for the observed alignment was automatically selected under the Bayesian Information Criterion (BIC, Schwarz, 1978) among a set of 468 substitution models. The model assumed LG (Le and Gascuel, 2008) amino-acid replacement matrix with across sites rate heterogeneity modeled via FreeRate model (Soubrier et al., 2012) assuming six rate classes. Branch support values were inferred based on 1,000 bootstrap replicates employing an ultrafast bootstrap approximation (Hoang et al., 2018) as implemented in the IQ-TREE program. Branches were named as suggested by the Super-Nomenclature Committee for Grape Gene Annotation (sNCGGa) (Grimplet et al., 2014). A bootstrap value of 70 (recommended by the Committee) distinguished the genes within the majority of the classes. Whenever a branch containing both *Vitis* and *Arabidopsis* gene/genes was found in a subtree, a subclass was defined. The remaining *Vitis* genes were named independently. Moreover, different members of a subclass were distinguished by a letter and different splicing variants had the same name but followed by a number. In the few cases in which the direct hortolog from Arabidopsis was found, both names were retained.

## Results

### Downy Mildew Resistance and Polyphenol Content

**Figure 1:**
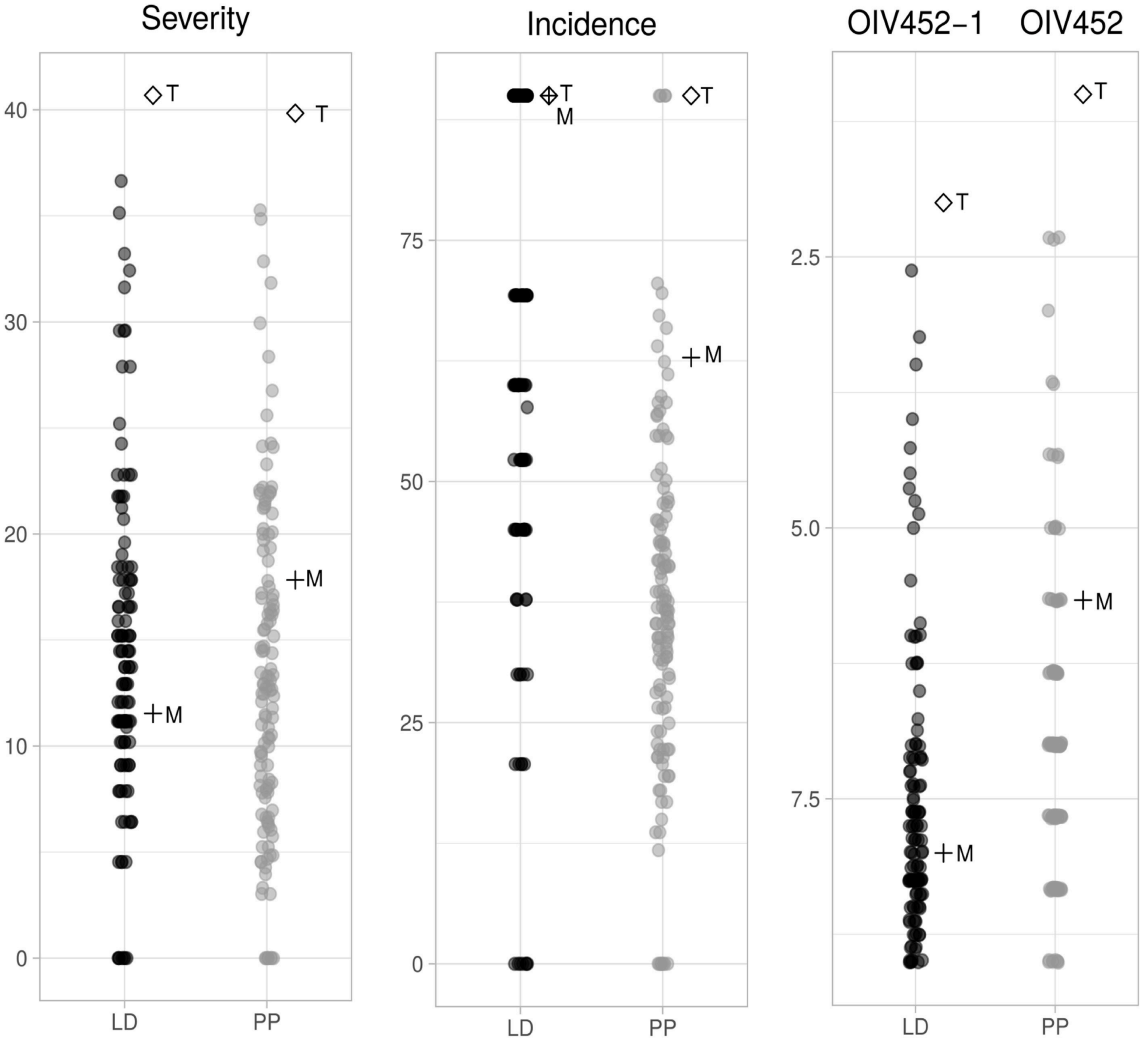
Phenotypic distribution of the three different parameters associated with resistance to *P. viticola* in the M×T progeny. Severity and incidence are expressed in percentage while the OIV descriptor follows a discrete scale from 1 to 9. These parameters were scored at 6 days post-inoculation (dpi) for leaf disks (LD) and at 8 dpi for potted plants (PP). The data were collected in June and August 2012 for the segregating population and in June and August 2013 for the two parental lines. The reproducibility of the two experiments was tested (*p* ≤ 0.05) on data collected in August 2012 and 2013 for the same genotypes as shown in Figure S1. M, “Merzling”; T, “Teroldego”.

*P. viticola* inoculation was performed on the entire progeny (2012) and on the two parental lines along with 11 repeated F1 individuals (2013). The reproducibility of both LD and PP experiments between the 2 years was verified by *t*-test on the three phenotypic parameters (Figures S1A,B) and it confirmed an overall coherence between 2012 and 2013 infection both for LD and PP. Altogether the phenotypic results indicated an approximately normal distribution of severity, incidence and OIV452(−1) in the progeny and confirmed a mid-resistant and susceptible phenotype for “Merzling” and “Teroldego,” respectively. It is relevant to underline that several F1 individuals were transgressive with respect to the resistance donor “Merzling” exhibiting a higher level of resistance, while none resulted more susceptible than the parental “Teroldego” (Figure 1).

**Figure 2:**
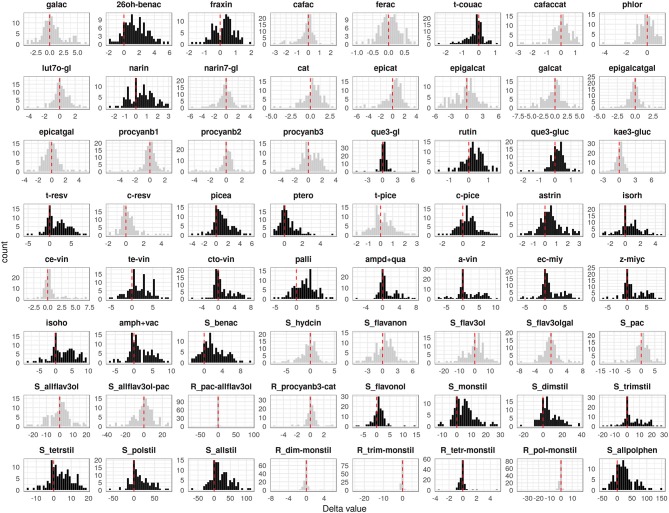
Distribution of the difference between *P. viticola*-inoculated (PI) and mock-inoculated (MI) values (delta) associated with 64 polyphenol-related parameters recorded in the M×T progeny. Black distributions highlight significantly modulated compounds. The meaning of the parameter abbreviations is reported in Table S1B.

Analogously to the DM resistance, the polyphenol-related data—the measurement of 64 parameters on PP of 11 repeated F1 individuals (Figure S2)—performed between 2012 and 2013 were found to be reproducible (*p* ≤ 0.05, corrected for multiplicity applying a Bonferroni correction). The differences turned out to be significant (*p* ≤ 0.05) in 13 out of 64 cases, exhibiting good reproducibility overall. Moreover, in many cases (e.g., 2,6-DHBA, fertaric acid, *cis*-piceid, *cis*-resveratrol, pterorostilbene, *cis*-ε-viniferin) the range of metabolic content identified in the progeny was greater than in the parents, suggesting a transgressive segregation (Figure S3). A significant delta value between PI and MI plants (*p* ≤ 0.05, corrected for multiplicity applying a Bonferroni correction) was detected for 32 different value distributions, each representing variation in the content of polyphenols: 22 of stilbenoid class, out of which seven monomeric (*trans*-resveratrol, piceatannol, pterostilbene, *cis*-piceid, astringin, isorhamnetin, and the sum of monomeric stilbenoids), five dimeric (*trans*-ε-viniferin, *cis*/*trans* ω-viniferin, pallidol, ampelopsin D+quadrangularin A, and the sum of dimers), four trimeric (α-viniferin, E-*cis*-miyabenol C, Z-miyabenol C, and the sum of trimers), three tetrameric (isohopeaphenol, ampelopsin H + vaticanol C-like isomer, and the sum of tetramers), the ratio between tetramers and monomers, the sum of polymers and all stilbenoids; two of benzoic acid class (2,6-DHBA and the sum of benzoic acids); the coumarin fraxin; the t-coutaric hydroxycinnamic acid; the flavanone naringenin; four flavonols (quercetin-3-glucoside, rutin, quercetin-3-glucuronide, and the sum of flavonols); and finally the sum of all polyphenols (Figure 2).

### QTL Mapping

#### The M×T Linkage Map

**Figure 3:**
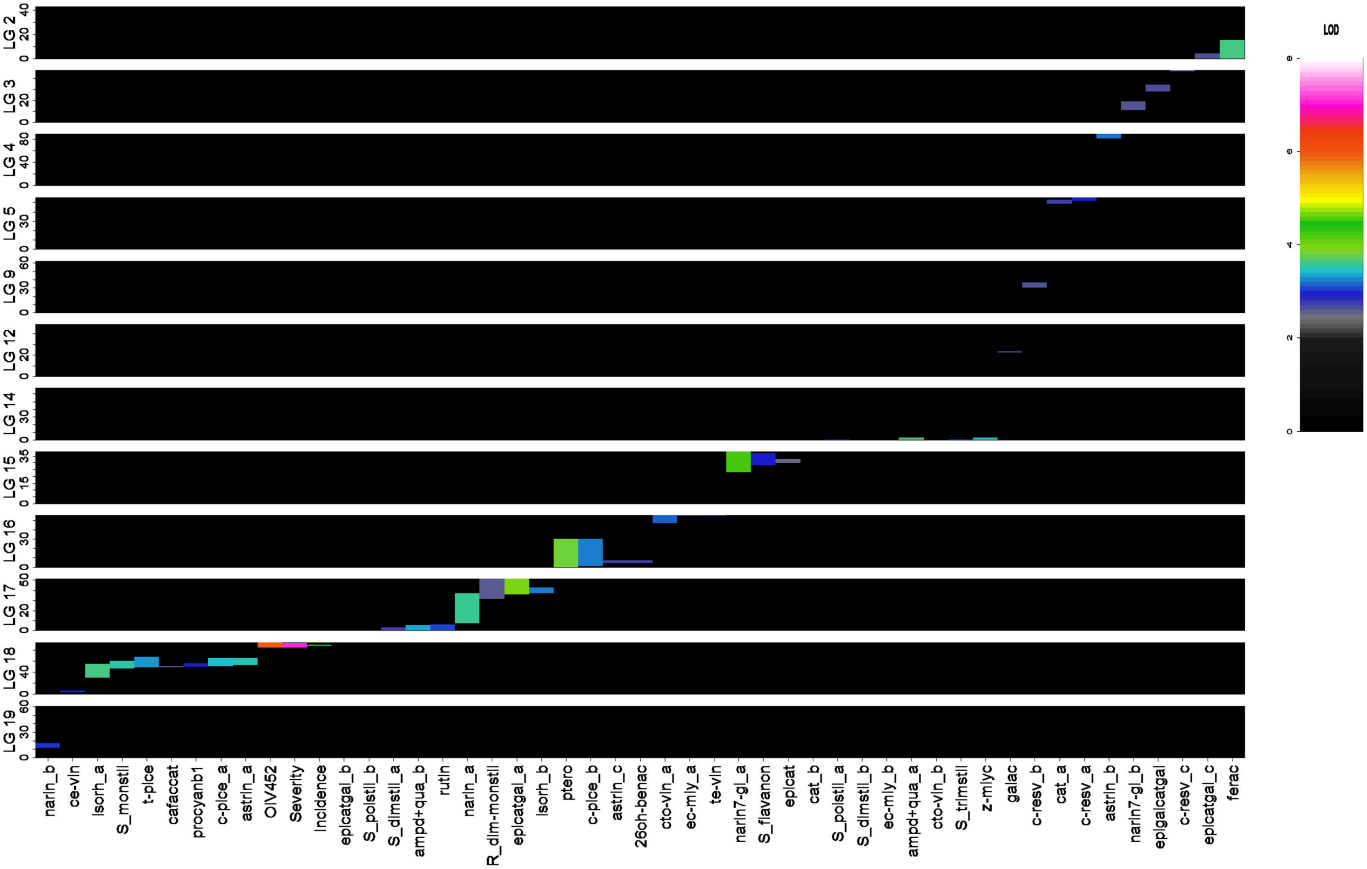
QTL distribution across the 19 M × T LGs. The meaning of the parameter abbreviations is reported in Table S1.

Out of 136 initial putative full-sib individuals, 129 resulted to be true-to-type F1 individuals upon the genotyping analysis. Of these, three were discarded because they presented >20% missing data (progeny information in Table S4). Out of the 192 scored, 181 markers were ordered into 19 LGs allowing the construction of the M×T consensus map. Marker order was generally consistent between parental and consensus homolog LGs, with local inversion of tightly linked markers, and reflected the backbone of previous published maps (Salmaso et al., 2008; Vezzulli et al., 2008). The remaining 11 markers consisted of one unlinked and 10 showing distorted segregation ratios with a probability *p* ≤ 0.05. The distribution of the 181 mapped markers into different segregation types showed that 91.1% allowed discrimination between paternal and maternal inherited allele. The total length of the consensus map was 1,162.7 cM with a mean distance between adjacent markers of 6.4 cM (Figure S4). The overall linkage map statistics are reported in Table S4. This map was finally employed in a QTL mapping survey to identify putative genomic regions involved in the genetic control of DM resistance and polyphenol variation upon *P. viticola* inoculation; a total of 49 significant QTLs associated with 33 different parameters were detected on 12 LGs (Figure 3, Table 1).

**Table 1:** List of the 49 identified significant QTLs associated to three DM resistance and 30 polyphenol-related parameters.

**Trait**	**Class**	**Parameter**	**Experiment**	**LG**	**LOD threshold (α = 5)**	**LOD peak**	**LOD peak position (cM)**	**LOD peak offset (bp)**	**% Expl Var**	**Close-to-QTL-start SSR**	**Close-to-QTL-end SSR**
Downy mildew resistance	–	Severity	Leaf Disks	18	2.7	5.73	89.312	26483989	19.9	UDV737	UDV737
			Potted Plants	18	2.8	7.12	88.312	24699030	22.9	VVIN16	UDV737
		Incidence	Leaf Disks	18	2.8	4.33	89.312	26483989	15.4	UDV737	UDV737
			Potted Plants	18	2.8	3.18	88.312	24699030	11.0	VVIN16	UDV737
		OIV 452-1	Leaf Disks	18	2.7	4.17	89.312	26483989	14.9	UDV737	UDV737
		OIV 452	Potted Plants	18	2.7	5.99	88.312	24699030	19.7	VVIN16	UDV305
Polyphenol content	Hydroxycinnamic acids	ferac	Potted Plants	2	2.4	3.68	0.000	2349171	13.2	VVIB01	VMC6F1
		cafaccat	Potted Plants	18	2.6	2.64	50.020	10721189	9.6	VVCS1H	VVCS1H
	Benzoic acids	galac	Potted Plants	12	2.5	2.69	22.593	6174660	9.8	VCHR12A	VCHR12A
		26oh-benac	Potted Plants	16	2.5	2.78	6.388	9994586	10.1	UDV104	UDV009
	Flavanones	narin	Potted Plants	17	2.6	3.64	23.182	3562640	13	VVIQ22-2	VMC9G4
				19	2.6	3.07	14.411	2124093	11.1	UDV023	UDV023
		narin7-gl	Potted Plants	15	2.5	4.28	30.709	16280816	15.2	VMC5G8	VMC8G3-2
				3	2.5	2.65	14.000	1817755	9.7	VMC8F10	VMC8F10
		S_flavanon	Potted Plants	15	2.4	2.99	30.709	16292317	10.8	VVIV24	VMC4D9-2
	Flavan-3-ol monomers and dimers	cat	Potted Plants	5	2.7	2.79	50.351	20620536	10.1	VMC9B5	VMC9B5
				15	2.4	2.52	30.709	15943274	9.2	VVIM42-2	VMC4D9-2
		epicat	Potted Plants	15	2.4	2.57	30.709	16292317	9.4	VVIV24	VMC4D9-2
		epigalcatgal	Potted Plants	3	2.5	2.70	30.471	4136318	9.9	UDV021	VMC1A5
		epicatgal	Potted Plants	17	2.5	4.02	44.395	8038301	14.3	VVIB09	VVIP16
				18	2.7	2.80	93.424	24868000	10.2	UDV305	UDV305
				2	2.5	2.68	0.000	2349171	9.8	VVIB01	VVIB01
		procyanb1	Potted Plants	18	2.9	2.99	53.134	12072631	10.9	VVCS1H	VVIM10
	Flavonol	rutin	Potted Plants	17	2.5	3.10	2.000	3033023	11.2	VMC3C11-1	VMC2H3
	Monomeric stilbenoids	c-resv	Potted Plants	5	2.6	2.99	55.224	24864769	10.8	VMC4C6	VMC4C6
				9	2.5	2.69	34.482	15302997	9.8	VCHR9A	VCHR9A
				3	2.5	2.57	47.034	11617504	9.4	VVMD28	VVMD28
		ptero	Potted Plants	16	2.5	3.89	16.522	9583955	13.9	UDV013	SC80189026
		t-pice	Potted Plants	18	2.8	3.37	56.134	13374598	12.1	VVCS1H	VVIP08
		c-pice	Potted Plants	18	2.9	3.48	55.134	12175152	12.5	VVIM10	VVIP08
				16	2.5	3.28	6.388	4092596	11.8	UDV104	SC80189026
		astrin	Potted Plants	18	2.8	3.53	57.134	12138633	12.7	VVIP08	VVIP08
				4	2.6	3.26	88.528	23105311	11.8	VMC6G10	VMC6G10
				16	2.4	2.78	6.000	9826198	10.1	UDV104	UDV009
		isorh	Potted Plants	18	2.8	3.69	49.020	9521743	13.2	VCHR18A	VVIM10
				17	2.5	3.29	39.977	9113940	11.8	VVIB09	VVIB09
		S_monstil	Potted Plants	18	2.8	3.58	54.134	12506620	12.8	VVCS1H	VVIM10
	Polymeric stilbenoids	ce-vin	Potted Plants	18	2.8	3.02	5.715	3362208	10.9	VMC8B5	VMC8B5
		te-vin	Potted Plants	16	2.6	2.74	55.429	21844881	10	SCU14	SCU14
		cto-vin	Potted Plants	16	2.6	3.18	51.154	21027861	11.5	VMC5A1	SCU14
				14	2.8	2.94	0.000	1413666	10.7	VMCNG1E1	VMCNG1E1
		ampd+qua	Potted Plants	14	2.7	3.66	0.000	1413666	13.1	VMCNG1E1	VMCNG1E1
				17	2.6	3.42	0.000	2165045	12.3	VMC3C11-1	SCU06
		ec-miy	Potted Plants	16	2.5	2.65	55.429	21844881	9.7	SCU14	SCU14
				14	2.6	2.64	0.000	1413666	9.6	VMCNG1E1	VMCNG1E1
		z-miyc	Potted Plants	14	2.8	3.52	0.000	1413666	12.6	VMCNG1E1	VMCNG1E1
		S_dimstil	Potted Plants	17	2.4	2.79	0.000	2165045	10.2	VMC3C11-1	VMC3C11-1
				14	2.7	2.69	0.000	1413666	9.8	VMCNG1E1	VMCNG1E1
		S_trimstil	Potted Plants	14	2.7	3.09	0.000	1413666	11.2	VMCNG1E1	VMCNG1E1
		S_polstil	Potted Plants	14	2.6	2.95	0.000	1413666	10.7	VMCNG1E1	VMCNG1E1
				17	2.7	2.73	0.000	2165045	9.9	VMC3C11-1	VMC3C11-1
		R_dim-monstil	Potted Plants	17	2.1	2.64	45.395	8472290	9.6	VMC9G4	VVIP16

#### The DM Resistance QTLs

Regarding the phenotypic data associated with DM resistance recorded on PP at 6 dpi, the first QTL identified was related to the severity parameter, showing a maximum LOD value of 7.12 (22.9% of explained variance), and located along the distal arm of chromosome 18 (88.3 cM). A co-localized QTL resulted associated with the OIV 452 descriptor with an LOD peak of 5.99 (19.7% of explained variance). Concerning the incidence parameter, the detected QTL was slightly shifted and showed a maximum LOD value of 3.18 (11% of explained variance). These results based on PP phenotypic data were confirmed by the results obtained on LD where a lower LOD peak was calculated per each parameter, except for disease incidence, which is more sensitive to the intra-plant leaf variability (Table 1, Figure 3). This overall genomic interval, ranging from 22.6 to 27.8 Mb of LG 18, co-localizes with the known *Rpv3* locus which is characterized by the flanking UDV305 and UDV737 SSR markers (Di Gaspero et al., 2012). Given their genetic profile in this work, the haplotype *Rpv3* ^*null*−271^ was identified as associated with DM resistance in “Merzling” and named *Rpv3-3*, according to the rules established by the international grapevine research community (www.vitaceae.org; http://www.vivc.de/). Not reliable, confirmed twice between PP and LD experiments, minor QTLs were also identified (data not show).

#### The Polyphenol-Related QTLs

As concerning polyphenol variation, significant QTLs were newly detected for a total of 30 parameters, including both the original and the derived ones. LOD peak values spanned from 2.52 to 4.28 (Table 1, Figure 3).

Overall, considering the QTL physical distribution, four clusters were located on LGs 18, 17, 16, and 15 by abundance order. In particular, a comprehensive cluster of QTLs positioned on LG 18 was associated with caffeic acid+catechin condensation, five parameters related to monomeric stilbenoids, *cis*-ε-viniferin, epicatechin gallate and procyanidin B1, and did not result overlapping with the distal region associated with DM resistance (Figure 3).

Regarding hydroxycinnamic acids, one QTL associated with fertaric acid (LOD peak value of 3.68) and one to caffeic acid+catechin condensation (LOD peak value of 2.64) were located on LGs 2 and 18, explaining, respectively 13.2 and 9.6% of the total phenotypic variance. In terms of benzoic acids, gallic acid, and 2,6-DHBA were correspondingly mapped on LGs 12 and 16, with 9.8 and 10.1% of explained variance.

Within the group of monomeric stilbenoids, three QTLs were found to be associated with *cis*-resveratrol on LGs 5, 9, and 3, explaining 10.8, 9.8, and 9.4% of total variance, respectively. A QTL for pterostilbene was located on LG 16 with a LOD peak value of 3.89, which corresponded to 13.9% of explained variance. Two QTLs, positioned on LGs 16 and 18, were related to the *cis*-piceid content (11.8 and 12.5% of explained variance), while *trans*-piceid was mapped only on the LG 18 region which explained a similar variance. Astringin was under control of three genomic regions (LGs 16, 4, and 18) which corresponded to a range from 10.1 to 12.7% of the total phenotypic variance. Two QTLs associated with isorhapontin were positioned on LGs 18 and 17, with 13.2 and 11.8% of explained variance. Finally, a QTL corresponding to the region controlling the content of all the monomeric stilbenoids mentioned above was found on LG 18 for the sum of monomeric stilbenoids. Concerning polymeric stilbenoids, *cis*-ε-viniferin was mapped on LG 18, in a region upstream to the one associated with monomeric stilbenoids and encompassing 10.9% of the total phenotypic variance; indeed, a QTL related to the *trans*-ε-viniferin content, explaining a highly similar variance, was detected on LG 16 in a region far from the one associated with the monomeric stilbenoids and coincident with the one explaining the 9.7% of the variance of E-*cis*-miyabenol content described below. *Cis*+*trans*-ω-viniferin was under control of two genomic regions (LGs 16 and 14) which corresponded to the 11.5 and 10.7% of the total phenotypic variance. In particular, 85% of the LG 16 region was specifically associated with this specific dimer. Two genomic regions associated with ampelopsin D+quadrangularin A were identified on LGs 14 and 17; these QTLs showed a LOD peak value of 3.66 and 3.42, with 13.1 and 12.3% of explained variance, respectively. Two QTLs, positioned on LGs 16 and 14, were related to the E-*cis*-miyabenol content (9.7 and 9.6% of explained variance), while Z-miyabenol C was mapped only on the LG 14 region which explained 12.6% of the total phenotypic variance. Considering derived parameters, two QTLs were identified on LGs 17 and 14 for the sum of dimeric stilbenoids (a mean of 10% of explained variance), whereas the sum of trimeric stilbenoids was associated only with the QTL on LG 14, explaining 11.2% of the total phenotypic variance. For total polymeric stilbenoids, including tetrameric stilbenoids, the two QTLs identified on LGs 14 and 17 were confirmed, with 10.7 and 9.9% of explained variance. Finally, a QTL was detected as associated with the ratio of dimeric to monomeric stilbenoids on a different region of LG 17, explaining 9.6% of the total variance. This region was also controlling the isorhapontin content as previously highlighted. Contrary to LG17, on LG14 the region associated with all the parameters previously described was coincident.

Within the flavanon group, two QTLs related to the naringenin content were found on LGs 17 and 19 (LOD peak value of 3.64 for the major QTL), with 13 and 11.1% of explained variance, respectively. The 56% of the first and the 100% of the second region were specifically associated with naringenin. Indeed, naringenin-7-glucoside was mapped on LGs 15 and 3 (LOD peak value of 4.28 for the major QTL), which respectively explained 15.2 and 9.7 of the total phenotypic variance. In particular, the 72% of the region on LG 13 was specific for this flavanon. For total flavanones, only the QTL located on LG 15 was shared, with 10.8% of explained variance. Regarding flavan-3-ol monomers and dimers, for catechin two QTLs were detected on LGs 5 and 15—of which the first one was private—explaining 10.1 and 9.1% of the total phenotypic variance, respectively. Two QTLs, the first one positioned on LGs 15 coincidently with the region associated with catechin and the second one on LG 3, were respectively related to the epicatechin and epigallocatechin gallate content, explaining a similar percentage of the total phenotypic variance. Three genomic regions associated with epicatechin gallate were identified on LGs 17, 18, and 2; these QTLs spanned from a LOD peak value of 4.02 to 2.68, with a corresponding range of explained phenotypic variance from 14.3 to 9.8%. Procyanidin B1 was mapped on LG 18 with 10.9% of explained variance. Finally, a QTL was associated with the flavonol rutin on LG 17, which explain 11.2% of the total phenotypic variance.

### Relationship Among Genetic Background, Resistance Level, and Polyphenol Induction

**Figure 4:**
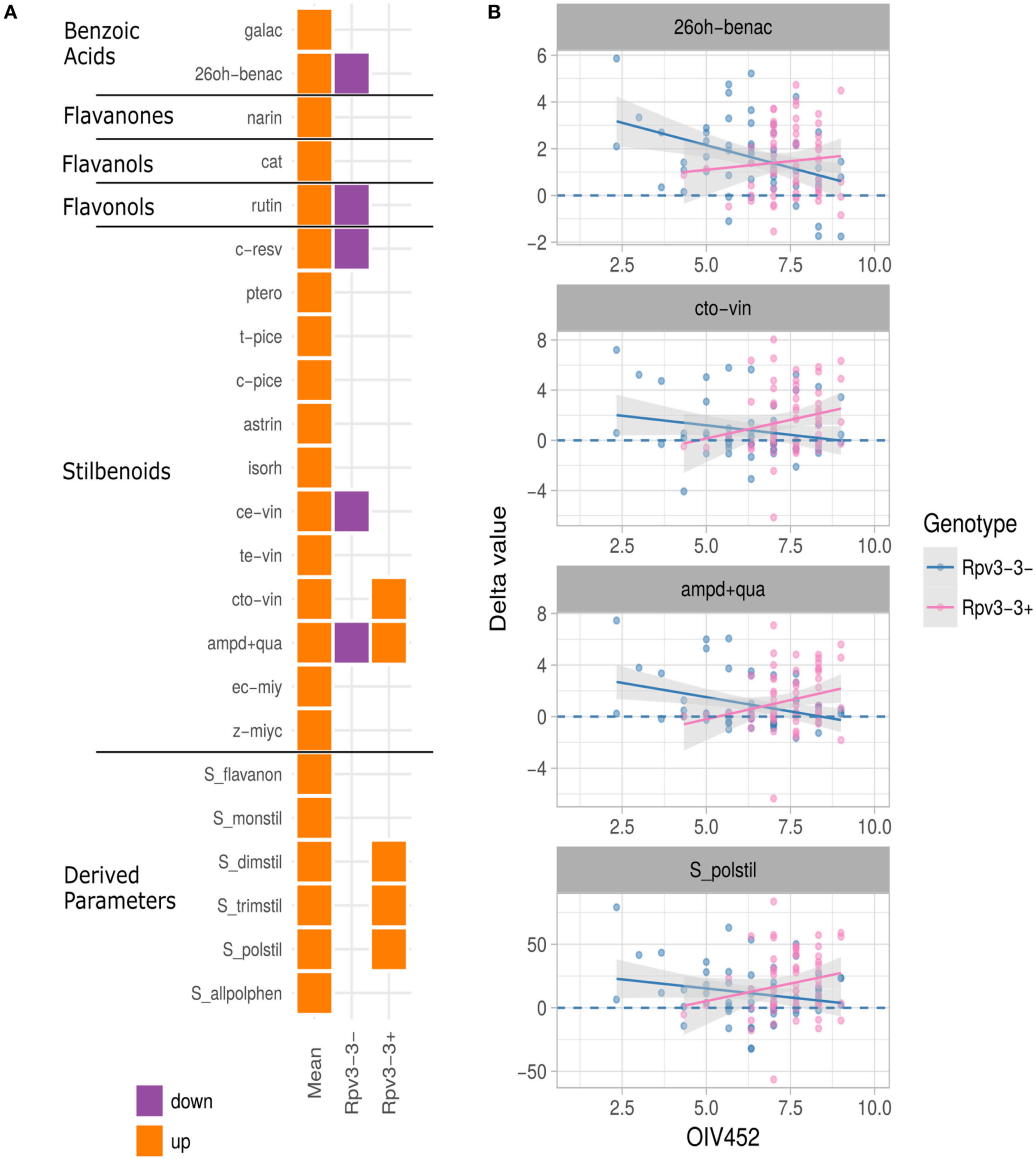
**(A)** Summary of univariate analysis performed on the 30 polyphenol-related parameters—with a QTL region associated—measured in the M × T progeny. The plot summarizes the outcomes of a series of regression analyses performed to assess the presence of a significant induction (*p* ≤ 0.05) and its dependence upon the resistance level (measured with the OIV 452 descriptor) for *Rpv3-3*^+^ and *Rpv3-3*^−^ genotypes. Orange and violet colors highlight up and down regulation, respectively. The first column (Mean) indicates the average value of the parameter distribution in the M×T progeny. **(B)** Examples of the univariate regression analyses. The meaning of the parameter abbreviations is reported in Table S1B.

Of the 30 polyphenolic compounds with an associated QTL, infection significantly affected production of 23. Out of these, synthesis of four compounds was induced in *Rpv3-3*^+^ genotypes only, synthesis of another four significantly decreased in *Rpv3-3*^−^ genotypes only, and one (ampelopsin D+quadrangularin A) was significantly induced and repressed in both *Rpv3-3*^+^ and *Rpv3-3*^−^ genotypes, respectively (Figure 4A).

Considering the *Rpv3-3* haplotype, the OIV 452 parameter and the polyphenol delta values, a relationship was highlighted exclusively for the stilbenoids. For instance, *cis*/*trans*-ω-viniferin and polymeric stilbenoids, on average induced in the progeny, resulted significantly induced in *Rpv3-3*^+^ genotypes showing a high OIV 452 value. By contrast, ampelopsin D+quadrangularin A, on average induced in the progeny as well, showed an opposite profile between *Rpv3-3*^+^ and *Rpv3-3*^−^ genotypes with high OIV 452 values. In addition, the induction of 2,6-DHBA, observed on average in the progeny, was very low in *Rpv3-3*^−^ genotypes with a high OIV 452 value (Figure 4B). In the *Rpv3-3*^−^ resistant genotypes no compound synthesis was significantly induced by the fungus (data not shown).

### Candidate Gene Identification and Characterization

#### Candidate Genes Underlying DM Resistance and Polyphenol-Related QTLs

The number of genes identified within each QTL region was extremely variable from a minimum of 5 (LG 18) to a maximum of 984 (LG 16). Due to the high number of GPs underlying the QTLs associated with the 33 assessed parameters, four different main criteria were adopted to select CGs for trait regulation (e.g., Figure S5 for enrichment analysis results). Upon this selection (Table S5), a refined list of the 95 most relevant unique CGs was generated, of which a few were previously characterized as involved in DM response and in the regulation of polyphenol synthesis (the so called “reference genes”), while the majority were newly identified. CGs mainly referred to the functional categories of signaling, secondary metabolism, regulation of transcription, and response to abiotic and biotic stimulus (Table 2).

**Table 2:** List of the 95 most relevant candidate genes associated with DM resistance and polyphenol content.

**Gene ID**		**Description**	**Functional category**	**Criteria of selection**	**Reference**	**Gene name**
**Severity (LG 18)**						
1	VIT_218s0117g00590	Laccase	Single reactions	Enriched	This work	
2	VIT_218s0117g00600	Laccase	Single reactions	Enriched	This work	
3	VIT_218s0117g00610	low quality protein: laccase-14-like	Single reactions	Enriched	This work	
4	VIT_218s0117g00625	low quality protein: laccase-14-like	Single reactions	Enriched	This work	
**Incidence (LG 18)**						
5	VIT_218s0041g01620	R protein L6 (TMV resistance protein N-like)	Biotic stress response	LOD max peak	This work	
**2,6-diOH-benzoic acid (LG 16)**						
6	VIT_216s0013g01920	Ser/Thr protein kinase	Protein kinase	Enriched, closed to LOD max offset	This work	
7	VIT_216s0013g01940	Kinase-like protein TMKL1	Protein kinase	Enriched, closed to LOD max offset	This work	
8	VIT_216s0013g01990	CLL1B clavata1-like receptor like K (RLK)	Protein kinase	Enriched, closed to LOD max offset, reference gene	This work	
9	VIT_216s0013g02130	Protein kinase	Protein kinase	Enriched, closed to LOD max offset	This work	
10	VIT_216s0013g02170	Protein kinase	Protein kinase	Enriched, closed to LOD max offset	This work	
11	VIT_216s0013g01390	Receptor kinase homolog LRK10	Protein kinase	Enriched, closed to LOD max offset	This work	
12	VIT_216s0013g00900	Ethylene-responsive TF ERF105	Ethylene Signaling	Enriched, reference gene	This work	
13	VIT_216s0013g01070	Ethylene-responsive TF ERF105	Ethylene Signaling	Enriched, reference gene	This work	
14	VIT_216s0013g01570	Myb domain protein 92	Regulation of transcription	Category of interest	This work	VvMYB194
**Rutin**						
15	VIT_217s0000g02660	MYBC2-L2	Regulation of transcription	Category of interest, reference gene	Cavallini et al., 2015	VvMYBC2-L2
16	VIT_217s0000g02710	Myb domain protein 4R1	Regulation of transcription	Category of interest	This work	
17	VIT_217s0000g02730	Myb domain protein 4R1	Regulation of transcription	Category of interest	This work	
***Cis*****-resveratrol**						
18	VIT_205s0094g00480	Ethylene-responsive protein	Ethylene Signaling	Category of interest		
19	VIT_209s0018g00240	WRKY40 like	Regulation of transcription	Category of interest	Corso et al., 2015	VvWRKY28
20	VIT_209s0018g00300	N-acetyltransferase hookless1 HLS1	Ethylene Signaling	Category of interest		
21	VIT_203s0132g00040	ATHVA22A	ABA Signaling	Enriched		
22	VIT_203s0132g00050	ATHVA22A	ABA Signaling	Enriched		
23	VIT_203s0132g00080	ATHVA22A	ABA Signaling	Enriched		
24	VIT_203s0132g00090	ATHVA22A	ABA Signaling	Enriched		
25	VIT_203s0132g00100	ATHVA22A	ABA Signaling	Enriched		
26	VIT_203s0097g00700	Pathogenesis-related protein 1 (PRP 1)	Jasmonate salicylate signaling	Category of interest		
***Cis*****-piceid (LG=16)**						
12	VIT_216s0013g00900	Ethylene-responsive TF ERF105	Ethylene Signaling	Enriched, reference gene	This work	
13	VIT_216s0013g01070	Ethylene-responsive TF ERF105	Ethylene Signaling	Enriched, reference gene	This work	
27	VIT_216s0100g00750	Stilbene synthase	Phenylpropanoid metabolism	Enriched	This work	VvSTS7
28	VIT_216s0100g00760	Stilbene synthase	Phenylpropanoid metabolism	Enriched	This work	VvSTS8
29	VIT_216s0100g00770	Stilbene synthase	Phenylpropanoid metabolism	Enriched	This work	VvSTS9
30	VIT_216s0100g00780	Stilbene synthase	Phenylpropanoid metabolism	Enriched	This work	VvSTS10
31	VIT_216s0100g00800	Stilbene synthase	Phenylpropanoid metabolism	Enriched	This work	VvSTS12
32	VIT_216s0100g00810	Stilbene synthase	Phenylpropanoid metabolism	Enriched	This work	VvSTS13
33	VIT_216s0100g00830	Stilbene synthase	Phenylpropanoid metabolism	Enriched	This work	VvSTS15
34	VIT_216s0100g00840	Stilbene synthase	Phenylpropanoid metabolism	Enriched	This work	VvSTS16
35	VIT_216s0100g00850	Stilbene synthase	Phenylpropanoid metabolism	Enriched	This work	VvSTS17
36	VIT_216s0100g00860	Stilbene synthase	Phenylpropanoid metabolism	Enriched	This work	VvSTS18
37	VIT_216s0100g00880	Stilbene synthase	Phenylpropanoid metabolism	Enriched	This work	VvSTS19
38	VIT_216s0100g00900	Stilbene synthase	Phenylpropanoid metabolism	Enriched	This work	VvSTS20
39	VIT_216s0100g00910	Stilbene synthase	Phenylpropanoid metabolism	Enriched, reference gene	Höll et al., 2013	VvSTS21
40	VIT_216s0100g00920	Stilbene synthase	Phenylpropanoid metabolism	Enriched, reference gene	Höll et al., 2013	VvSTS22
41	VIT_216s0100g00930	Stilbene synthase	Phenylpropanoid metabolism	Enriched	this work	VvSTS23
42	VIT_216s0100g00940	Stilbene synthase	Phenylpropanoid metabolism	Enriched	This work	VvSTS24
43	VIT_216s0100g00950	Stilbene synthase	Phenylpropanoid metabolism	Enriched	This work	VvSTS25
44	VIT_216s0100g00960	Stilbene synthase	Phenylpropanoid metabolism	Enriched	This work	VvSTS26
45	VIT_216s0100g00990	Stilbene synthase	Phenylpropanoid metabolism	Enriched, reference gene	Höll et al., 2013	VvSTS27
46	VIT_216s0100g01000	Stilbene synthase	Phenylpropanoid metabolism	Enriched, reference gene	Höll et al., 2013	VvSTS28
47	VIT_216s0100g01010	Stilbene synthase	Phenylpropanoid metabolism	Enriched, reference gene	Höll et al., 2013	VvSTS29
48	VIT_216s0100g01020	Stilbene synthase	Phenylpropanoid metabolism	Enriched	This work	VvSTS30
48	VIT_216s0100g01020	Stilbene synthase	Phenylpropanoid metabolism	Enriched	This work	VvSTS31
49	VIT_216s0100g01040	Stilbene synthase	Phenylpropanoid metabolism	Enriched	This work	VvSTS32
50	VIT_216s0100g01060	Stilbene synthase	Phenylpropanoid metabolism	Enriched	This work	VvSTS33
51	VIT_216s0100g01070	Stilbene synthase	Phenylpropanoid metabolism	Enriched	This work	VvSTS35
52	VIT_216s0100g01100	Stilbene synthase	Phenylpropanoid metabolism	Enriched	This work	VvSTS36
53	VIT_216s0100g01110	Stilbene synthase	Phenylpropanoid metabolism	Enriched	This work	VvSTS37
54	VIT_216s0100g01120	Stilbene synthase	Phenylpropanoid metabolism	Enriched	This work	VvSTS39
55	VIT_216s0100g01130	Stilbene synthase	Phenylpropanoid metabolism	Enriched, reference gene	Höll et al., 2013	VvSTS41
56	VIT_216s0100g01140	Stilbene synthase	Phenylpropanoid metabolism	Enriched	This work	VvSTS42
57	VIT_216s0100g01150	Stilbene synthase	Phenylpropanoid metabolism	Enriched	This work	VvSTS43
58	VIT_216s0100g01160	Stilbene synthase	Phenylpropanoid metabolism	Enriched, reference gene	Höll et al., 2013	VvSTS45
59	VIT_216s0100g01170	Stilbene synthase	Phenylpropanoid metabolism	Enriched, reference gene	Vannozzi et al., 2012	VvSTS46-VvSTS47-VvSTS48
***Trans*****-piceid (LG=18)**						
60	VIT_218s0001g12900	JA O-methyltransferase	Amino acid metabolism	JA signaling	This work	
61	VIT_218s0001g13110	Peroxidase	Amino acid metabolism	Involved in oligomerization	This work	VviPrxIII34a
62	VIT_218s0001g15390	Gaiacol peroxidase	Amino acid metabolism	Involved in oligomerization	This work	VviPrxIII21a
**Pterostilbene (LG=16)**						
6	VIT_216s0013g01920	Ser/Thr protein kinase (PK)	Protein kinase	Enriched, closed to LOD max offset	This work	
7	VIT_216s0013g01940	Kinase-like protein TMKL1	Protein kinase	Enriched, closed to LOD max offset	This work	
8	VIT_216s0013g01990	CLL1B clavata1-like receptor like K (RLK)	Protein kinase	Enriched, closed to LOD max offset, reference gene	This work	
9	VIT_216s0013g02130	Protein kinase (PK)	Protein kinase	Enriched, closed to LOD max offset	This work	
10	VIT_216s0013g02170	Protein kinase (PK)	Protein kinase	Enriched, closed to LOD max offset	This work	
11	VIT_216s0013g01390	Receptor kinase homolog LRK10	Protein kinase	Enriched, closed to LOD max offset	This work	
12	VIT_216s0013g00900	Ethylene-responsive TF ERF105	Ethylene Signaling	Enriched, reference gene	This work	
13	VIT_216s0013g01070	Ethylene-responsive TF ERF105	Ethylene Signaling	Enriched, reference gene	This work	
27	VIT_216s0100g00750	Stilbene synthase	Phenylpropanoid metabolism	Enriched	This work	VvSTS7
28	VIT_216s0100g00760	Stilbene synthase	Phenylpropanoid metabolism	Enriched	This work	VvSTS8
29	VIT_216s0100g00770	Stilbene synthase	Phenylpropanoid metabolism	Enriched	This work	VvSTS9
30	VIT_216s0100g00780	Stilbene synthase	Phenylpropanoid metabolism	Enriched	This work	VvSTS10
31	VIT_216s0100g00800	Stilbene synthase	Phenylpropanoid metabolism	Enriched	This work	VvSTS12
32	VIT_216s0100g00810	Stilbene synthase	Phenylpropanoid metabolism	Enriched	This work	VvSTS13
33	VIT_216s0100g00830	Stilbene synthase	Phenylpropanoid metabolism	Enriched	This work	VvSTS15
34	VIT_216s0100g00840	Stilbene synthase	Phenylpropanoid metabolism	Enriched	This work	VvSTS16
35	VIT_216s0100g00850	Stilbene synthase	Phenylpropanoid metabolism	Enriched	This work	VvSTS17
36	VIT_216s0100g00860	Stilbene synthase	Phenylpropanoid metabolism	Enriched	This work	VvSTS18
37	VIT_216s0100g00880	Stilbene synthase	Phenylpropanoid metabolism	Enriched	This work	VvSTS19
38	VIT_216s0100g00900	Stilbene synthase	Phenylpropanoid metabolism	Enriched	This work	VvSTS20
39	VIT_216s0100g00910	Stilbene synthase	Phenylpropanoid metabolism	Enriched	This work	VvSTS21
40	VIT_216s0100g00920	Stilbene synthase	Phenylpropanoid metabolism	Enriched	This work	VvSTS22
41	VIT_216s0100g00930	Stilbene synthase	Phenylpropanoid metabolism	Enriched	This work	VvSTS23
42	VIT_216s0100g00940	Stilbene synthase	Phenylpropanoid metabolism	Enriched	This work	VvSTS24
43	VIT_216s0100g00950	Stilbene synthase	Phenylpropanoid metabolism	Enriched	This work	VvSTS25
44	VIT_216s0100g00960	Stilbene synthase	Phenylpropanoid metabolism	Enriched	This work	VvSTS26
45	VIT_216s0100g00990	Stilbene synthase	Phenylpropanoid metabolism	Enriched, reference gene	Höll et al., 2013	VvSTS27
46	VIT_216s0100g01000	Stilbene synthase	Phenylpropanoid metabolism	Enriched, reference gene	Höll et al., 2013	VvSTS28
47	VIT_216s0100g01010	Stilbene synthase	Phenylpropanoid metabolism	Enriched, reference gene	Höll et al., 2013	VvSTS29
48	VIT_216s0100g01020	Stilbene synthase	Phenylpropanoid metabolism	Enriched	This work	VvSTS30
48	VIT_216s0100g01020	Stilbene synthase	Phenylpropanoid metabolism	Enriched	This work	VvSTS31
49	VIT_216s0100g01040	Stilbene synthase	Phenylpropanoid metabolism	Enriched	This work	VvSTS32
50	VIT_216s0100g01060	Stilbene synthase	Phenylpropanoid metabolism	Enriched	This work	VvSTS33
51	VIT_216s0100g01070	Stilbene synthase	Phenylpropanoid metabolism	Enriched	This work	VvSTS35
52	VIT_216s0100g01100	Stilbene synthase	Phenylpropanoid metabolism	Enriched	This work	VvSTS36
53	VIT_216s0100g01110	Stilbene synthase	Phenylpropanoid metabolism	Enriched	This work	VvSTS37
54	VIT_216s0100g01120	Stilbene synthase	Phenylpropanoid metabolism	Enriched	This work	VvSTS39
55	VIT_216s0100g01130	Stilbene synthase	Phenylpropanoid metabolism	Enriched, reference gene	Höll et al., 2013	VvSTS41
56	VIT_216s0100g01140	Stilbene synthase	Phenylpropanoid metabolism	Enriched	This work	VvSTS42
57	VIT_216s0100g01150	Stilbene synthase	Phenylpropanoid metabolism	Enriched	This work	VvSTS43
58	VIT_216s0100g01160	Stilbene synthase	Phenylpropanoid metabolism	Enriched, reference gene	Höll et al., 2013	VvSTS45
59	VIT_216s0100g01170	Stilbene synthase	Phenylpropanoid metabolism	Enriched	Vannozzi et al., 2012	VvSTS46-VvSTS47-VvSTS48
63	VIT_216s0022g02470	Cationic peroxidase	Amino acid metabolism	Involved in oligomerization	This work	VviPrxIII08a
64	VIT_216s0100g00090	Cationic peroxidase	Amino acid metabolism	Involved in oligomerization	This work	VviPrxIII08b
65	VIT_216s0022g01690	Band 7 family (Hrp_c)	Auxiliary transport proteins		Casagrande et al., 2011	
66	VIT_216s0022g02040	PBS2 (PPHB susceptible 2)	Biotic stress response		Casagrande et al., 2011	
**Astringin (LGs=18, 16, 4)**						
60	VIT_218s0001g12900	JA O-methyltransferase	Amino acid metabolism	JA signaling	This work	
61	VIT_218s0001g13110	Peroxidase	Amino acid metabolism	Involved in oligomerization	This work	VviPrxIII34a
67	VIT_216s0013g01560	Myb domain protein 92	Regulation of transcription	Regulator	This work	VvMYB193
14	VIT_216s0013g01570	Myb domain protein 92	Regulation of transcription	Regulator	This work	VvMYB194
68	VIT_204s0023g03790	Jasmonate methyltransferase	Lipid metabolism	JA signaling	This work	
69	VIT_204s0023g03800	Jasmonate methyltransferase	Lipid metabolism	JA signaling	This work	
70	VIT_204s0023g03810	Jasmonate O-methyltransferase	Lipid metabolism	JA signaling	This work	
71	VIT_204s0044g01510	Histone deacetylase HDA14	Cell growth and death	LOD max peak	This work	
72	VIT_204s0044g01205	chitinase 1	Biotic stress response	Defense response	This work	
73	VIT_204s0023g03710	Myb domain protein 4B	Regulation of transcription	Regulator, reference gene	Cavallini et al., 2015	VvMYB4B
74	VIT_204s0044g01380	Myb domain protein 52	Regulation of transcription	Regulator	This work	
**Isorhapontin (LGs=18, 17)**						
75	VIT_218s0001g10450	VvAREB/ABF2	ABA Signaling	Category of interest	Nicolas et al., 2014	VvbZIP045
76	VIT_218s0001g11630	Allene oxide synthase	Lipid metabolism	reference gene	Casagrande et al., 2011	
60	VIT_218s0001g12900	JA O-methyltransferase (JMT)	Amino acid metabolism	Reference gene	Casagrande et al., 2011	
61	VIT_218s0001g13110	Peroxidase	Amino acid metabolism	Involved in oligomerization	This work	VviPrxIII34a
77	VIT_217s0000g07750	Peroxidase 65	Amino acid metabolism	Involved in oligomerization	This work	VviPrxIII15a
78	VIT_217s0000g07370	EDS1	Jasmonate salicylate signaling	Enriched	Casagrande et al., 2011	
79	VIT_217s0000g07375	EDS1	Jasmonate salicylate signaling	Enriched	Casagrande et al., 2011	
80	VIT_217s0000g07400	EDS1	Jasmonate salicylate signaling	Enriched	Casagrande et al., 2011	
81	VIT_217s0000g07420	EDS1	Jasmonate salicylate signaling	Enriched	Casagrande et al., 2011	
82	VIT_217s0000g07560	EDS1	Jasmonate salicylate signaling	Enriched	Casagrande et al., 2011	
***Cis*****-ε-viniferin (LG=18)**						
83	VIT_218s0001g02400	Laccase 14	Single reactions	Involved in oligomerization	This work	
84	VIT_218s0001g02410	Laccase/Diphenol oxidase family protein	Single reactions	Involved in oligomerization	This work	
***Cis*****+*****trans*****-ω-viniferin (LG=16*****)***						
85	VIT_216s0148g00300	Receptor serine/threonine kinase	Protein kinase	Enriched, reference gene	This work	
86	VIT_216s0098g00820	Peroxidase 3	Amino acid metabolism	Involved in oligomerization	This work	VviPrxIII23a
87	VIT_216s0098g00330	ORG1 (OBP3-responsive gene 1)	Jasmonate salicylate signaling	JA signaling	This work	
**Ampelopsin D+quadrangularin A (LG=17)**						
88	VIT_217s0000g02650	MYBC2-L4	Regulation of transcription	Regulator	Cavallini et al., 2015	VvMYBC2-L4
15	VIT_217s0000g02660	MYBC2-L2	Regulation of transcription	Regulator, reference gene	Cavallini et al., 2015	VvMYBC2-L2
16	VIT_217s0000g02710	Myb domain protein 4R1	Regulation of transcription	Regulator	This work	
17	VIT_217s0000g02730	Myb domain protein 4R1	Regulation of transcription	Regulator	This work	
**S_monomeric stilbenoids (LG=18)**						
61	VIT_218s0001g13110	Peroxidase	Amino acid metabolism	Involved in oligomerization	This work	VviPrxIII34a
62	VIT_218s0001g15390	Gaiacol peroxidase	Amino acid metabolism	Involved in oligomerization	This work	VviPrxIII21a
**S_dimeric stilbenoids**						
8	VIT_217s0000g02650	MYBC2-L4	Regulation of transcription	Regulator	Cavallini et al., 2015	VvMYBC2-L4
15	VIT_217s0000g02660	MYBC2-L2	Regulation of transcription	Regulator, reference gene	Cavallini et al., 2015	VvMYBC2-L2
16	VIT_217s0000g02710	Myb domain protein 4R1	Regulation of transcription	Regulator	This work	
17	VIT_217s0000g02730	Myb domain protein 4R1	Regulation of transcription	Regulator	This work	
**R_dimeric-monomeric stilbenoids (LG=17)**						
77	VIT_217s0000g07370	EDS1	Jasmonate salicylate signaling	Enriched	Casagrande et al., 2011	
78	VIT_217s0000g07375	EDS1	Jasmonate salicylate signaling	Enriched	Casagrande et al., 2011	
79	VIT_217s0000g07400	EDS1	Jasmonate salicylate signaling	Enriched	Casagrande et al., 2011	
80	VIT_217s0000g07420	EDS1	Jasmonate salicylate signaling	Enriched	Casagrande et al., 2011	
81	VIT_217s0000g07560	EDS1	Jasmonate salicylate signaling	Enriched	Casagrande et al., 2011	
89	VIT_217s0000g09070	Histone deacetylase HDA6	Jasmonate salicylate signaling	Enriched	This work	
77	VIT_217s0000g07750	Peroxidase 65	Amino acid metabolism	Involved in oligomerization	This work	VviPrxIII15a
**S_trimeric stilbenoids (LG=14)**						
90	VIT_214s0060g02280	C3HC4-type ring finger	Regulation of transcription	Regulator	This work	
**Z-miyabenol C (LG=14)**						
91	VIT_214s0060g01710	Ribosomal protein L18	Protein metabolism and modification	LOD max peak	This work	
**S_polymeric stilbenoids (LG=14)**						
92	VIT_214s0060g02420	JmjC domain-containing protein	Regulation of transcription	Regulator	This work	
93	VIT_214s0060g02440	Indeterminate(ID)-domain 2	Regulation of transcription	Regulator	This work	
94	VIT_214s0060g02640	Myb family	Regulation of transcription	Regulator	This work	
95	VIT_214s0060g02660	Nuclear transcription factor Y subunit B-5	Regulation of transcription	Regulator	This work	

*(i) The gray background indicates the genes for which expression was evaluated in 12 F1 individuals of the M × T progeny by qRT-PCR; (ii) genes associated with different parameters are repeated in the Table; (iii) Gene ID, code of 12Xv2 gene predictions as retrieved by CRIBI database (http://genomes.cribi.unipd.it/DATA/V2/); functional category: functional category at the second or third level of description*.

Besides disease-related (NBS-LRR) genes alone representing a significant part of the grapevine genome (Malacarne et al., 2012) and found also in high numbers within the major QTL on LG 18 associated with DM resistance parameters, four laccases (*VIT_218s0117g00590, VIT_218s0117g00600, VIT_218s0117g00610, VIT_218s0117g00625*), which can be part of the defense response due to pathogen recognition mediated by the LRR domain, drew our attention (Table S5).

Regarding the polyphenol content trait, we focused on CGs (i) underlying QTLs associated with polyphenols induced by infection and (ii) showing disparity between *Rpv3-3*^+^ and *Rpv3-3*^−^ genotypes characterized by a different level of DM resistance. Within the category of signaling, many selected genes belong to the kinase protein family, as well as to ethylene, ABA and JA signaling pathways. Two genes appear to be of special interest, one coding for the LRK10 Receptor kinase homolog (*VIT_216s0013g01390*) and the other encoding CLL1B clavata1-like receptor S/T protein kinase (*VIT_216s0013g01990*) located within the QTL region on LG16 linked to 2,6-DHBA and to pterostilbene. In addition, a gene coding for a Receptor serine/threonine kinase (*VIT_216s0148g00300*) resulted associated with *cis*-ω viniferin, as well as two genes encoding the Ethylene-responsive transcription factor ERF105 (*VIT_16s0013g00900* and *VIT_16s0013g01070*) were linked to 2,6-DHBA, pterostilene, and *cis*-piceid content. In addition, five genes (*VIT_203s0132g00040, VIT_203s0132g00050, VIT_203s0132g00080, VIT_203s0132g00090, VIT_203s0132g00100*), coding for a cluster of HVA22-like abscisic acid-induced proteins, were linked to *cis*-resveratrol content and the gene encoding the bZIP factor VvAREB/ABF2 (*VIT_218s0001g10450*) was exclusively associated to isorhapontin. Three JA O-methyltransferases (*VIT_204s0023g03790, VIT_204s0023g03800, VIT_204s0023g03810*) were associated with astringin, an additional JA O-methyltransferase (*VIT_218s0001g12900*) was related to astringin and isorhapontin, five genes (*VIT_217s0000g07370, VIT_217s0000g07375, VIT_217s0000g07400, VIT_217s0000g07420, VIT_217s0000g07560*) coding for EDS1 were linked to isorhapontin and the ratio between dimeric and monomeric stilbenoids, and finally one gene (*VIT_216s0098g00330*) encoding ORG1 was associated with the regulation of *cis*+*trans*-ω-viniferin content (Table 2).

A cluster of stilbene synthase genes, mostly not specifically related to DM response previously, was found in the QTL intervals associated with *cis*-piceid and pterostilbene. In addition, six peroxidase genes (herein named *VviPrxIII08a, VviPrxIII08b, VviPrxIII15a, VviPrxIII21a, VviPrxIII23a, VviPrxIII34a*, Figure 6) underlied monomeric and oligomeric stilbenoid-related QTL regions and two laccase genes (*VIT_218s0001g02400 and VIT_218s0001g02410*) were associated with ε-viniferin.

Two of the genes recently identified as encoding a set of R2R3-MYB C2 repressors of phenylpropanoid levels (Cavallini et al., 2015) were linked to the regulation of ampelopsin D+quadrangularin A, the sum of dimeric stilbenoids and rutin (*VvMYBC2-L2*) and to the regulation of *trans*-piceid and astringin content (*VvMYB4B*). Another seven *MYB* genes were identified: *VIT_216s0013g01560* (*VvMYB193* in Wong et al., 2016) and *VIT_216s0013g01570* (*VvMYB194* in Wong et al., 2016) in the region controlling both 2,6-DHBA and astringin content, *VIT_204s0044g01380* in the region controlling astringin content, *VIT_217s0000g02710* and *VIT_217s0000g02730* as associated with ampelopsin D+quadrangularin A, the sum of dimeric stilbenoids and rutin content, and finally *VIT_214s0060g02640* related to the sum of polymeric stilbenoids. Finally, a WRKY factor (precisely VvWRKY28 in Wang et al., 2014) was associated to *cis*-resveratrol content.

#### Newly Identified Genes Candidate to Stilbenoid Oligomerization

Among all CGs, further investigation was dedicated to a set of 11 genes by assessing their transcript level in a set of 12 F1 individuals. The objective was to get further evidence of the association between the identified genomic regions and the traits under investigation. These genotypes exhibited disparity in pathogen resistance level, stilbenoid-related parameters with an mQTL associated and haplotype status at the *Rpv3* locus (Figure S6). These genes encoded: (i) five out of six peroxidases identified within monomeric and oligomeric stilbenoid-related QTL regions (*VviPrxIII08a, VviPrxIII08b, VviPrxIII15a, VviPrxIII21a, VviPrxIII23a*); (ii) two of the six laccases described above (*VIT_218s0117g00590* and *VIT_218s0001g02400*), the first associated with disease severity and the other with ε-viniferin and therefore putatively involved in its oligomerization; (iii) three stilbene synthases (*VvSTS27-8-9, VvSTS41*, and *VvSTS48*) already associated with DM response (Vannozzi et al., 2012; Höll et al., 2013) and here related to the regulation of pterostilbene and *cis*-piceid content; (iv) one Histone deacetylase HDA14 (*VIT_204s0044g01510*) found in correspondence of the LOD peak offset of the QTL on LG 4 associated with astringin.

**Figure 5:**
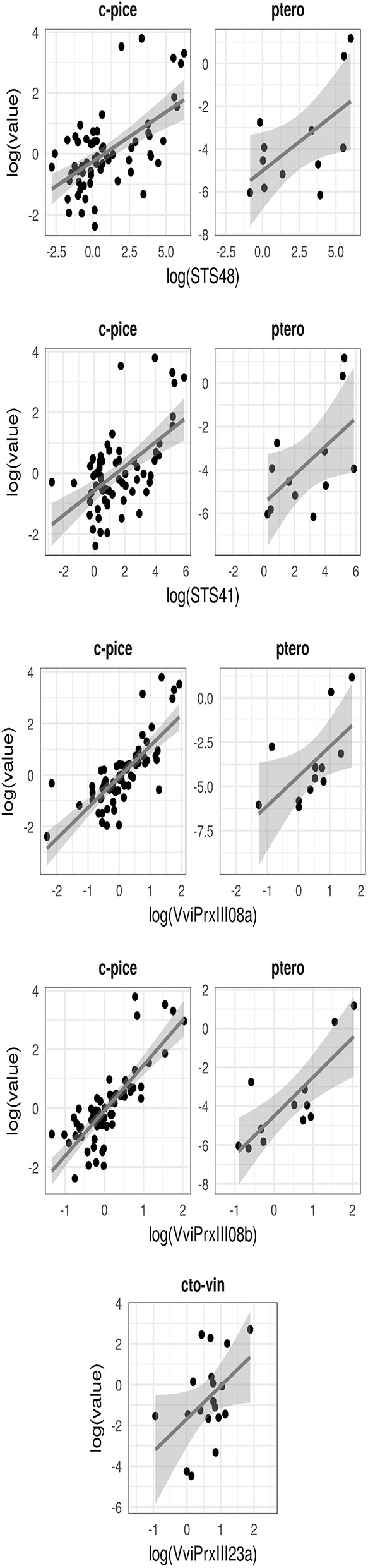
Regression analysis which highlights the association between metabolic induction and normalized relative expression of selected candidate genes in *Rpv3-3*^+^ and *Rpv3-3*^−^ selected genotypes. c-pice, *cis*-piceide; ptero, pterostilbene; cto-vin, *cis* + *trans*-ω-viniferin.

The gene expression study revealed a significant correlation between (i) the content of *cis*-piceid and pterostilbene and the expression level both of *VviPrxIII08a* and *VviPrxIII08b*, (ii) the content of ω-viniferin and the expression level of *VviPrxIII23a*, and finally (iii) the content of *cis*-piceid and pterostilbene and the expression level both of *VvSTS41* and *VvSTS48* at 6 dpi (Figure 5).

**Figure 6:**
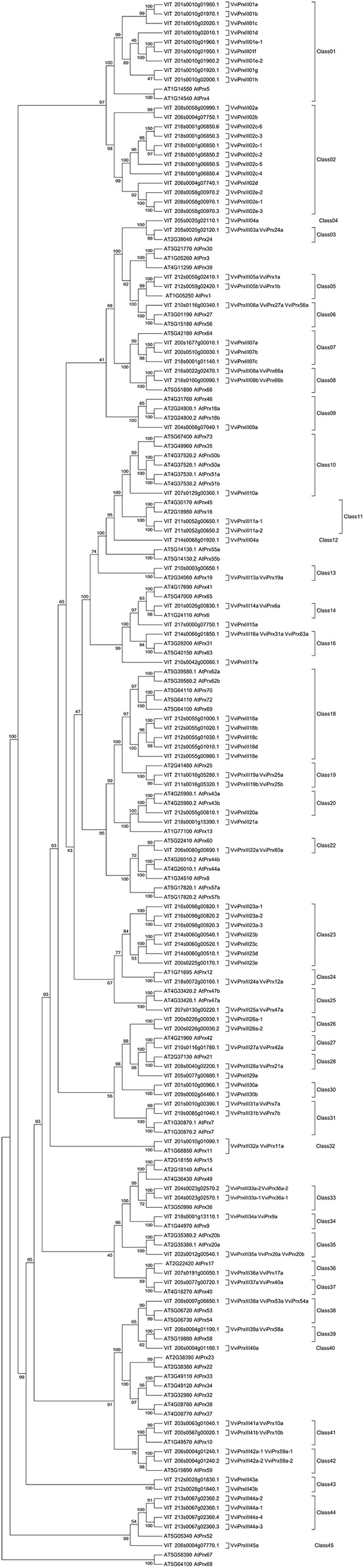
Phylogenetic analysis of the grapevine peroxidase gene family.

In order to gather additional information about the peroxidases found in the QTL and correlated with stilbenoid induction upon *P. viticola* infection we performed a phylogenetic analysis of the whole grapevine peroxidase gene family, which allowed us to attribute the 90 GPs to 45 different classes. The six peroxidases herein associated to stilbenoid metabolism are part of different classes: *VviPrxIII08a* and *VviPrxIII08b* belong to class 08 and are the putative orthologs of *AtPrx66, VviPrxIII15a* and *VviPrxIII21a* are the unique members of class 15 and 23, respectively, *VviPrxIII23a* has three different isoforms which belong to class 23 together with another four members, and finally *VviPrxIII34a* belongs to class 34 and is the putative ortholog of *AtPrx09* (Figure 6; Table S6).

## Discussion

### Characterization of the *Rpv3-3* haplotype

The M×T genetic map built in this study is an improved version in terms of progeny individuals and LG number as well as marker number/order compared to the one by Salmaso et al. (2008). The high percentage of markers (68%) that was mapped in both parents is close to the 62% of markers positioned into the reference grapevine genetic map, while the 5% of distorted markers is about half of the value previously observed (Adam-Blondon et al., 2004). This upgrading should result in visible improvement in the parental and consensus genetic maps to be suitable for further applications as the association to new phenotypes.

Herein, at the hypervariable microsatellite markers UDV305 and UDV737, we identified the haplotype null-271 as conferring resistance against *P. viticola*; this genetic variant is located on chromosome 18 and it derives on the “Merzling” side from the grand-parent “Seyval.” This haplotype was named *Rpv3-3* according to the VIVC nomenclature, given its co-localization with the *Rpv3* locus, which was subjected to selective sweep during grapevine breeding activities. In fact, a seminal study identified seven conserved haplotypes, which are overrepresented in grapevine breeding lines historically selected for DM resistance compared to their wild relatives, and are absent from the susceptible *V. vinifera* varieties (Di Gaspero et al., 2012). These genetic variants may carry *Rpv3* alleles or adjacent *Rpv3* paralogs that constantly remained linked with the diagnostic markers. Upon this reported association analysis, nowadays only two wild relative *Rpv3* haplotypes have been characterized in segregating populations. The *Rpv3-1* haplotype—preserved in the “Seibel 4614” lineage—was firstly identified in the German hybrid “Regent” (Welter et al., 2007; van Heerden et al., 2014) and in the Hungarian hybrid “Bianca” (Bellin et al., 2009) through QTL analyses. The second of these resistance haplotypes—named *Rpv3-2*, conserved in the “Munson” lineage—has recently been confirmed by QTL mapping (Zyprian et al., 2016). In this work, we have validated the *Rpv3-3* haplotype—derived from the descent group founder “Noah”—by means of a QTL analysis on the M×T progeny.

Given this outcome, we attempted the characterization of several genotypes related to Merzling (data not shown). Unlike the comprehensive *Rpv3* survey reported by Di Gaspero et al. (2012), we detected the *Rpv3-3* haplotype also in the “Merzling” offspring “Solaris,” so far known to carry only the *Rpv10* locus derived from *V. amurensis*. This was due to the masking effect of a different *Rpv3* haplotype (*Rpv3-1*) derived from the second resistance donor Gf.Ga-52-42 in the QTL study (Schwander et al., 2012). Analogously, besides the *Rpv 10* locus, we found that “Bronner” and “Cabernet Cortis” unexpectedly inherited the *Rpv3-3* haplotype from their parent “Merzling” and “Solaris,” respectively; all these genotypes used as parental lines demonstrated to transmit this acquired haplotype to their respective progenies (Vezzulli S., personal communication). Finally, herein this specific genetic variant was detected also in the related Baron, Prior, and Cabernet Cantor (data not shown). These findings confirmed that grapevine breeders traditionally selected genotypes with a certain degree of pyramiding, namely accumulation of more than one *R*-locus (Töpfer et al., 2011), suggesting a reinforcement role of the *Rpv3-3* haplotype on other *R*-locus effects. For this reason our investigation can be considered a “genetic” upgrade of the pioneer *Rpv3* study by Di Gaspero et al. (2012).

Genes belonging to the NBS-LRR superfamily were detected in the regions underlying QTLs associated with all disease resistance parameters, in agreement with the first studied *Rpv3* resistance haplotype encoding NB-LRR and LRR-kinase receptors (Di Gaspero and Foria, 2015). According to the Effector-Triggered Immunity model, *R* gene products sense the pathogen effectors and activate signal transduction pathways (Cui et al., 2015). In grapevine, *Rpv3*-dependent resistance follows this model of gene-for-gene interaction (Casagrande et al., 2011). In previous studies, the resistance haplotype was revealed to be necessary and sufficient to trigger a hypersensitive response (HR) leading to cell death in the proximity of sites infected by *P. viticola* (Bellin et al., 2009; Zyprian et al., 2016). In the current study, weakened or delayed HR was observed on LD of *Rpv3-3*^+^ genotypes (data not shown) with lower OIV452-1 values; this overall phenomenon can be due to the combination of different factors—high inoculum concentration and RH—leading to shorter incubation time, more infection sites and faster hyphal growth under highly conducive conditions (Gessler et al., 2011). In addition, it is relevant to highlight that the phenotypic distribution (ranges: OIV 452 from 1.6 to 9, Severity from 0 to 33.3%, Incidence from 0 to 100%) on PP revealed an average mid-resistance level of *Rpv3-3*^+^ genotypes, in agreement with the recent study by Foria et al. (2018) which shed light on various *Rpv3* haplotypes responsible for different disease resistance degrees. Finally, unlike the expectation in the case of an *R*-locus, the non-binomial distribution of each DM resistance parameter suggests that this trait is controlled by multiple factors in this genetic background. The M×T QTL study refers to the concept of Advanced Backcross-QTL (AB-QTL)—recently recovered in grapevine—which combines QTL analysis and variety development by designing a mapping/breeding scheme for the simultaneous identification and introgression of wild haplotypes. AB-QTL relies on segregating populations in which most of the wild-parent genome that donates the trait of interest has been purged in early segregating generations by phenotypic selection (Tanksley and Nelson, 1996). This is relevant to guarantee QTL stability once the associated markers are screened in derived breeding materials. In fact, favorable QTL alleles identified in early generations often disappear in later back-cross generations, once the modifier genes that have epistatic interactions with the beneficial QTL alleles are removed from highly *V. vinifera* genetic backgrounds (Di Gaspero and Foria, 2015). Herein, we did not actually face cases of susceptible individuals carrying the *Rpv3-3*^+^ haplotype, whereas we found a few *Rpv3-3*^−^ individuals displaying DM resistance. Since unreliable minor QTLs were identified in both the M × T consensus and the maternal genetic map (data not shown), and no intra-locus recombination was detected, this phenomenon has still to be elucidated.

### Survey of Stilbenoid-Associated Regions Among the Discovered Polyphenol-Related QTLs

In the present study 46 novel metabolic (m)QTLs associated with 30 phenolics-related parameters were discovered. Among the new mQTLs, more than half were associated with stilbenoid-related parameters (monomers, dimers, and trimers), two with hydroxycinnamic acids, two with benzoic acids, five with flavanones, and eight with flavan-3-ol monomers and dimers. Pleiotropic effects, i.e., single gene producing multiple effects on various traits, were recorded. Except for few cases encompassing different classes (e.g., ratio between dimeric and monomeric stilbenoids and naringenin), pleiotropy was detected along genomic intervals associated with parameters falling into the same class, such as monomeric stilbenoids and flavanones. Moreover, epistatic effects have recently been considered in many studies as relevant for complex traits, such as polyphenol biosynthesis. Epistasis, i.e., an additive-by-additive interaction between QTLs, assayed in populations segregating for an entire genome, has been found at a frequency close to that expected by chance alone (Bocianowski, 2013). Therefore, we cannot exclude epistatic effects in the case of characters controlled by more than one region, such as *cis*-piceid, astringin, and naringenin.

Although few research studies on proanthocyanidin and flavonol berry composition have recently been attempted in grapevine (Huang et al., 2012; Malacarne et al., 2015), most polyphenols do not have a known QTL associated. The mQTLs here identified fill this gap and reveal polyphenols with central role in *P. viticola*-grapevine interaction. In particular, this analysis allowed the identification of mQTLs associated with 17 different stilbenoid-related parameters, therefore representing a thorough characterization of stilbenoid regulation upon *P. viticola* infection on leaves.

A cluster of stilbene synthase genes, mostly not specifically related to DM response previously, was identified in the QTL intervals associated with *cis*-piceid and pterostilbene. Previous works revealed different patterns of transcript accumulation between the different *VvSTS* family members (Dai et al., 2012; Vannozzi et al., 2012; Höll et al., 2013; Shi et al., 2014) depending on the high variability in their regulatory regions (Chialva et al., 2018). Moreover, six interesting peroxidase and two laccase genes were identified as associated with stilbenoids therefore representing good candidates for ROS-mediated stilbene oligomerization (Calderón et al., 1994; Barceló et al., 2003; Pezet et al., 2004b). Indeed, there is some evidence supporting the enzymatic biotransformation of stilbenes by plant peroxidases (Takaya et al., 2005; Wan et al., 2011) and/or fungal laccases (e.g., Pezet et al., 1991; Breuil et al., 1998, 1999). It is still a debate if the oligomerization is driven by plant or fungal laccases, which catalyze the degradation of stilbene monomers allowing the fungus to escape from the action of grapevine phytoalexins. By phylogenetic reconstruction of the entire grapevine peroxidase gene family, we determined that *VviPrxIII08a* and *VviPrxIII08b* are the putative orthologs of *AtPrx66* involved in lignification (Tokunaga et al., 2009), *VviPrxIII34a* is the putative ortholog of *AtPrx09* which is closed to Arabidopsis members involved in lignin biosynthesis (Tognolli et al., 2002; Herrero et al., 2013), while *VviPrxIII15a, VviPrxIII21a*, and *VviPrxIII23a* have no orthologs in Arabidopsis. A validation of the results obtained at the metabolic level came from a transcriptional investigation in a set of 12 F1 individuals of the progeny, which highlighted a significant association between some monomeric and dimeric stilbenoids and the transcript level of three newly identified peroxidases besides known stilbene synthases.

The time dependent regulation of different inducible stilbenes upon abiotic and biotic stresses was reported to be at least transcriptional (e.g., Vannozzi et al., 2012; Höll et al., 2013; Wong et al., 2016) and coordinated by the action of both MYB and WRKY transcription factors (Malacarne et al., 2018; Vannozzi et al., 2018; Jeandet et al., 2019; Jiang et al., 2019). In the present work additional *MYB* genes were associated with stilbenoid formation; in particular, the known R2R3-MYB C2 repressors of phenylpropanoid levels (Cavallini et al., 2015) were located along the regions on LG4 and LG17 controlling some monomeric and dimeric stilbenoids, respectively. In addition, it is worth noting that the WRKY factor VvWRKY28 already found co-expressed with *VvSTS* transcripts in root and leaves (Corso et al., 2015) was here associated with *cis*-resveratrol content extending the list of WRKY factors identified as involved in the regulation of stilbenoid metabolism. It has recently been shown that the expression level of several members of the stilbene synthase gene family and genes responsible of the oxidative polymerization of phenolic compounds in the phenylpropanoid pathway is highly influenced by the “location” variable in a G×E interaction study (Dal Santo et al., 2018).

Our results showed that ABA and SA/JA signaling play an important role in the regulation of 2,6-DHBA, reported to be the de-activated form of SA (Bellés et al., 2006; Campos et al., 2014; Nawrocka et al., 2018), and of stilbenoid synthesis. A cluster of five genes encoding HVA22-like abscisic acid-induced proteins, belonging to a class of ABA- and stress-inducible proteins (Chen et al., 2002), was identified on LG3 associated with *cis*-resveratrol. Moreover, the transcriptional regulator VvbZIP045, recently characterized by Nicolas et al. (2014) as a key factor activating down-stream genes of the ABA signaling cascade during the grape berry ripening process, was here associated with the regulation of isorhapontin content. It should be noted that (Wang et al., 2018) have recently showed that *Muscadinia rotundifolia* “Noble” defense response to *P. viticola* infection is mediated by stilbene accumulation induced by ABA and SA phytohormones. Moreover, two genes (*VIT_216s0013g00900* and *VIT_216s0013g01070*) encoding Ethylene-responsive transcription factor ERF105, known to regulate the plant response to abiotic and biotic stress (Mizoi et al., 2012; Mishra et al., 2015), were selected within the 2,6 DHBA, *cis*-piceid, and pterostilbene associated regions. Remarkable was also the significant enrichment of protein kinases in the region on LG16 associated both with 2,6 DHBA and pterostilbene: two genes encoding respectively for CLV1 and LRK10, which are receptor-like kinases (RLK) previously related to plant-microbe interaction and stress responses (Shiu and Bleecker, 2001), and four calcium-dependent protein kinases involved in the translation of pathogen signal-induced changes in the Ca^2+^ concentration during plant defense reactions (Schulz et al., 2013).

### Combination of *Rpv3-3* Haplotype, Stilbenoid Induction, and DM Resistance

In our previous study we found evidence of a putative involvement of stilbenoids in conferring DM resistance in the M × T segregating population (Malacarne et al., 2011), without taking into account the genetic background. To shed light into the found *Rpv3*-mediated DM resistance mechanism we have investigated the genetic bases of this mechanism. Although no overalapping QTLs controlling both DM resistance and stilbenoid-related traits were detected, we could highlight a significant induction of most stilbenoids, especially the polymeric ones, in a high fraction of the individuals. To date several studies have reported on (i) stilbenoid induction upon *P. viticola* infection (e.g., Langcake and Pryce, 1976; Alonso-Villaverde et al., 2011), (ii) a more rapid and extensive accumulation of stilbenoids in DM-resistant vs. DM-susceptible genotypes (*V. vinifera* cultivars) (Chitarrini et al., 2017), (iii) an antifungal activity carried out by stilbenoids on *P. viticola* and other fungi (e.g., Pezet et al., 2004a; Adrian and Jeandet, 2012; Gabaston et al., 2017). What still remains to be elucidated is the defense mechanism underlying DM resistance conferred by different *Rpv* loci. This is crucial for pyramiding various DM resistance mechanisms, derived from different sources, in the process of genetic improvement.

A deeper look into the *Rpv3-3* haplotype within the M×T population highlighted a clear link among genetic background, DM resistance, and stilbenoid induction upon *P. viticola* infection. Unlike the *Rpv3-3*^−^ genotypes, the *Rpv3-3*^+^ showed on average a significant induction of two dimeric and one trimeric stilbenoids and a positive correlation between the extent of induction and DM resistance. A clear explanation of the resistance in the *Rpv3-3*^−^ individuals should be further elucidated, since in the present work we could not find any minor reliable DM resistance QTLs, as well as any metabolite exclusively induced in this group of genotypes.

## Data Availability

All datasets generated for this study are included in the manuscript and/or the Supplementary Files.

## Author Contributions

SV conceptualized the project, carried out genetic mapping and QTL analysis, and drafted the manuscript. GM conceptualized the project, performed the candidate gene selection with related expression analysis, and drafted the manuscript. DM and ZHM carried out the metabolite analysis. UV supported the metabolite analysis. AV and LZ performed the phenotyping assays. MS supported the phenotyping assays. CD and EB carried out the genotyping analysis. RV supported the genotyping analysis. VG performed the phylogenetic study and contributed to the manuscript revision. PF performed the statistical analysis. CM conceptualized as well as coordinated the project, and contributed to the discussion of the results. All authors read and approved the final manuscript.

### Conflict of Interest Statement

The authors declare that the research was conducted in the absence of any commercial or financial relationships that could be construed as a potential conflict of interest.
