# Supplementary figures and images for: The Rpv3-3 Haplotype and Stilbenoid Induction Mediate Downy Mildew Resistance in a Grapevine Interspecific Population
Source: Front Plant Sci. 2019 Mar 6;10:234. doi: 10.3389/fpls.2019.00234 (PMC6414455; doi:10.3389/fpls.2019.00234)

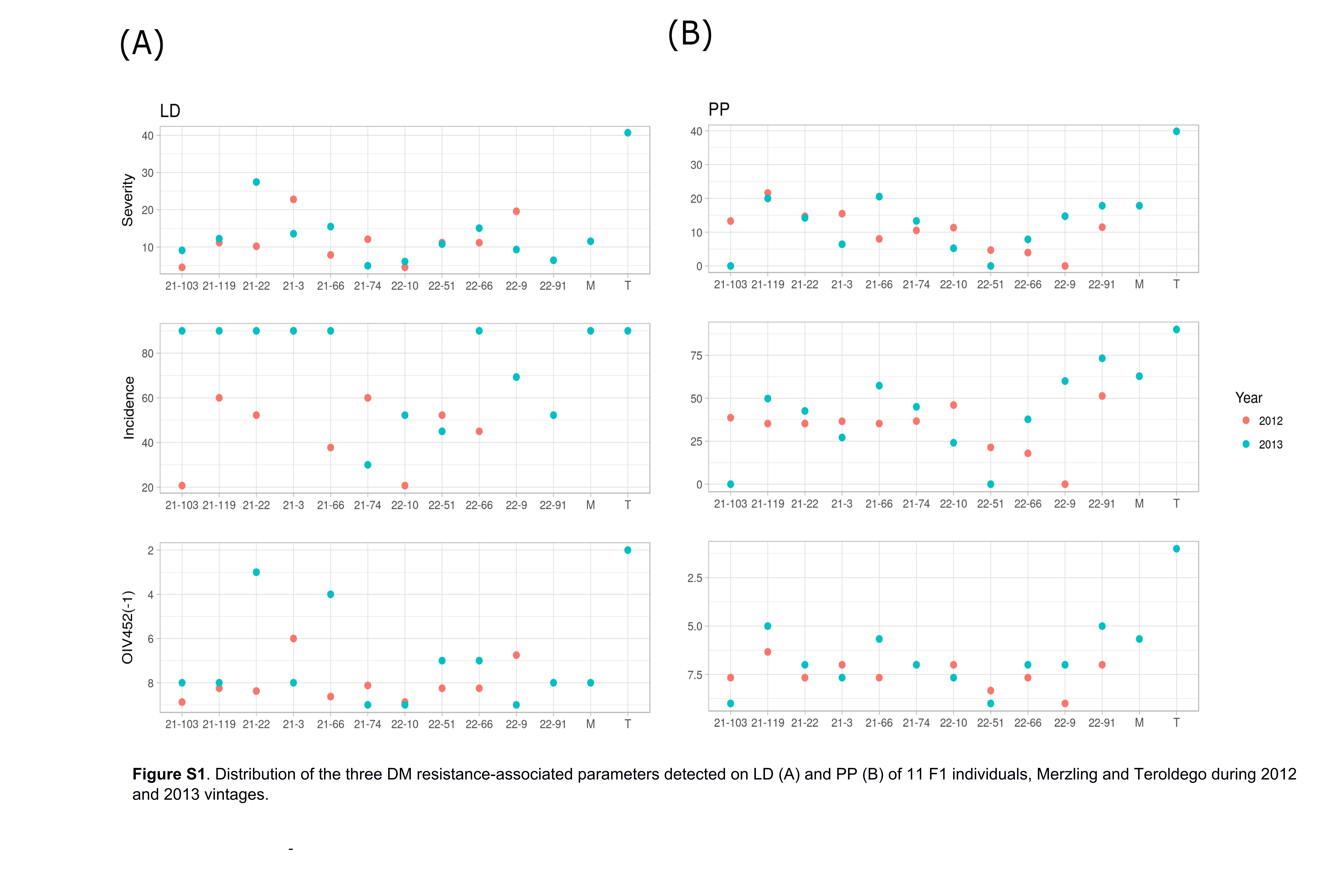

Supplement: Supplementary file 1 [file Image_1.TIF]

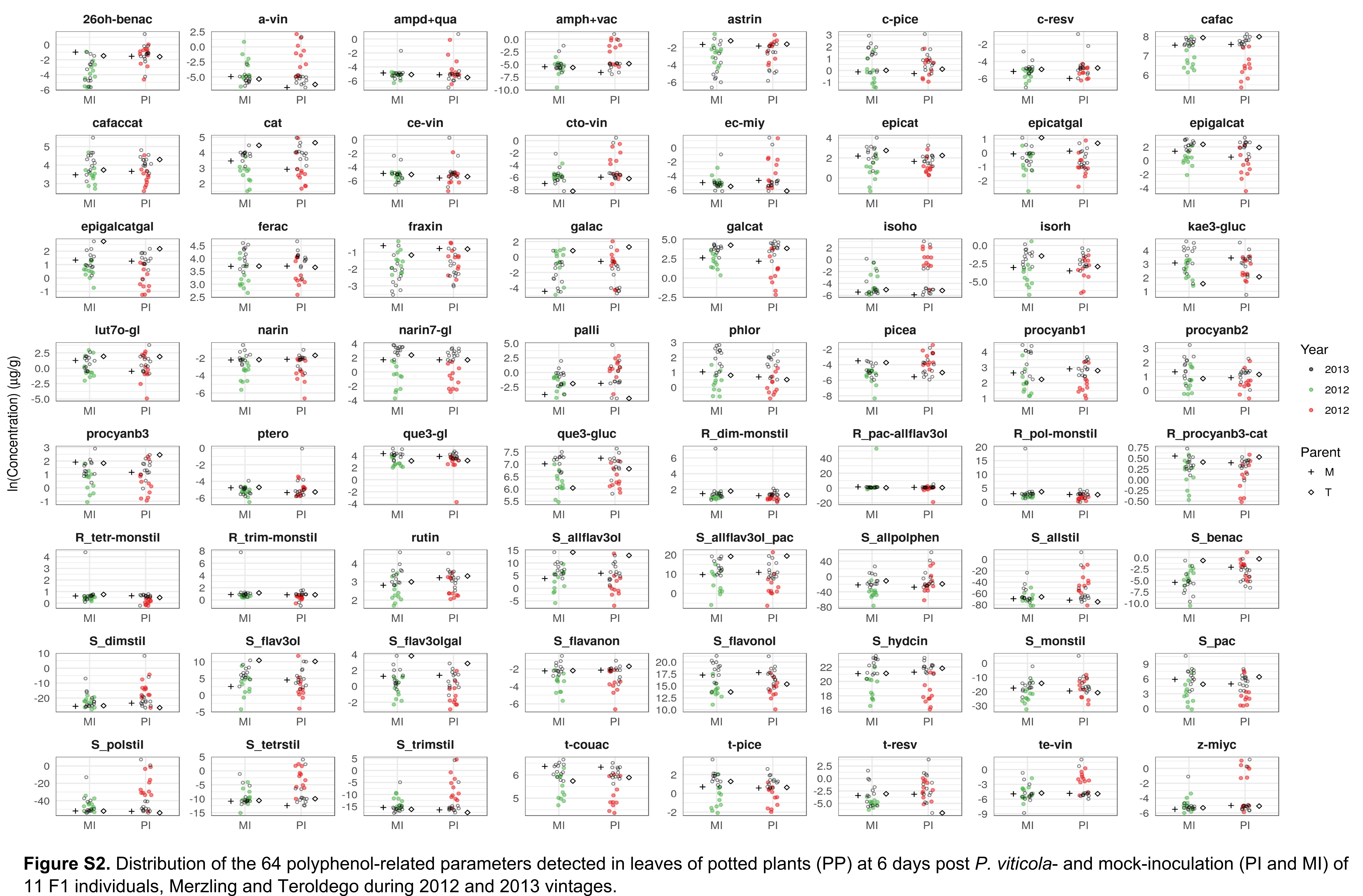

Supplement: Supplementary file 2 [file Image_2.TIF]

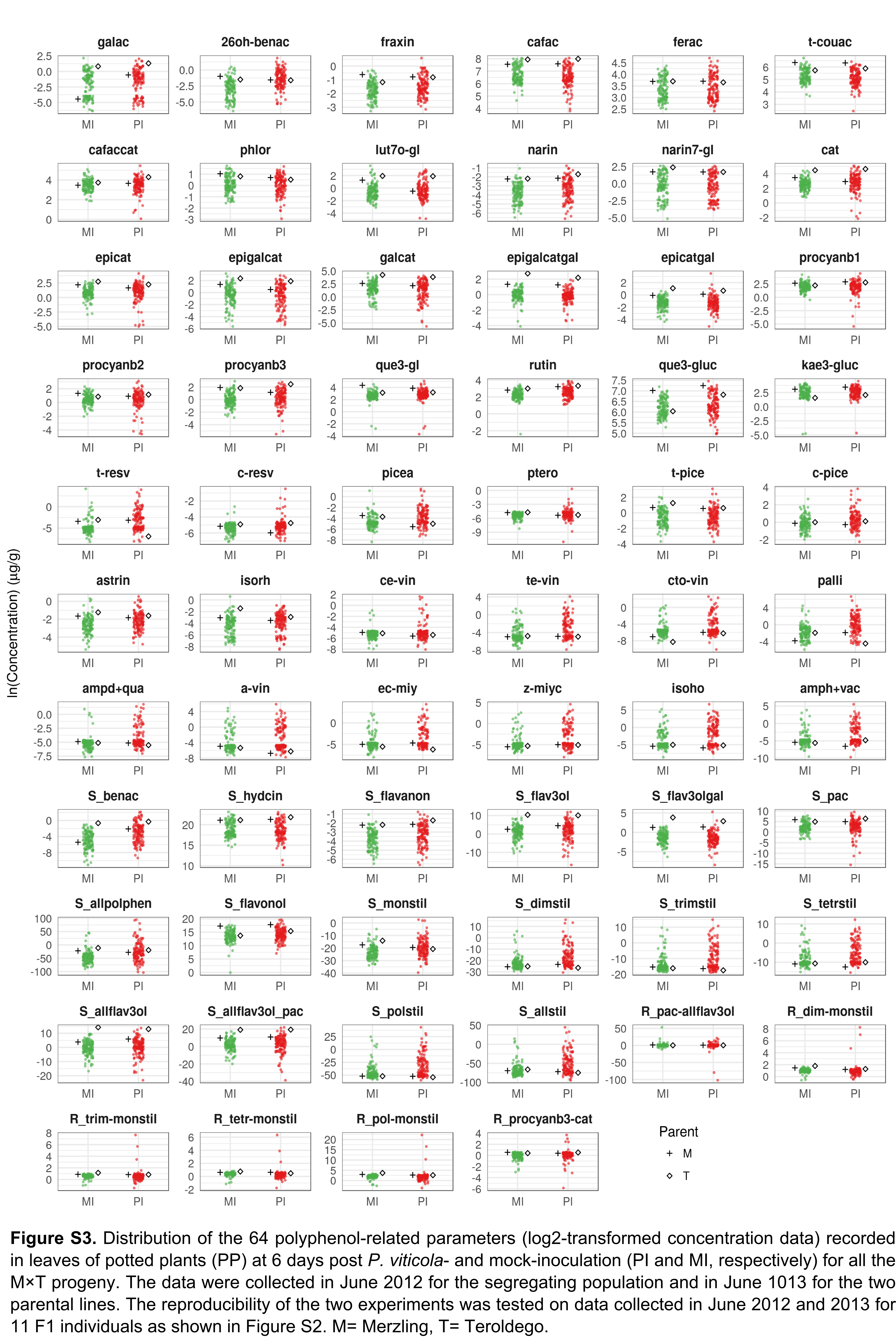

Supplement: Supplementary file 3 [file Image_3.TIF]

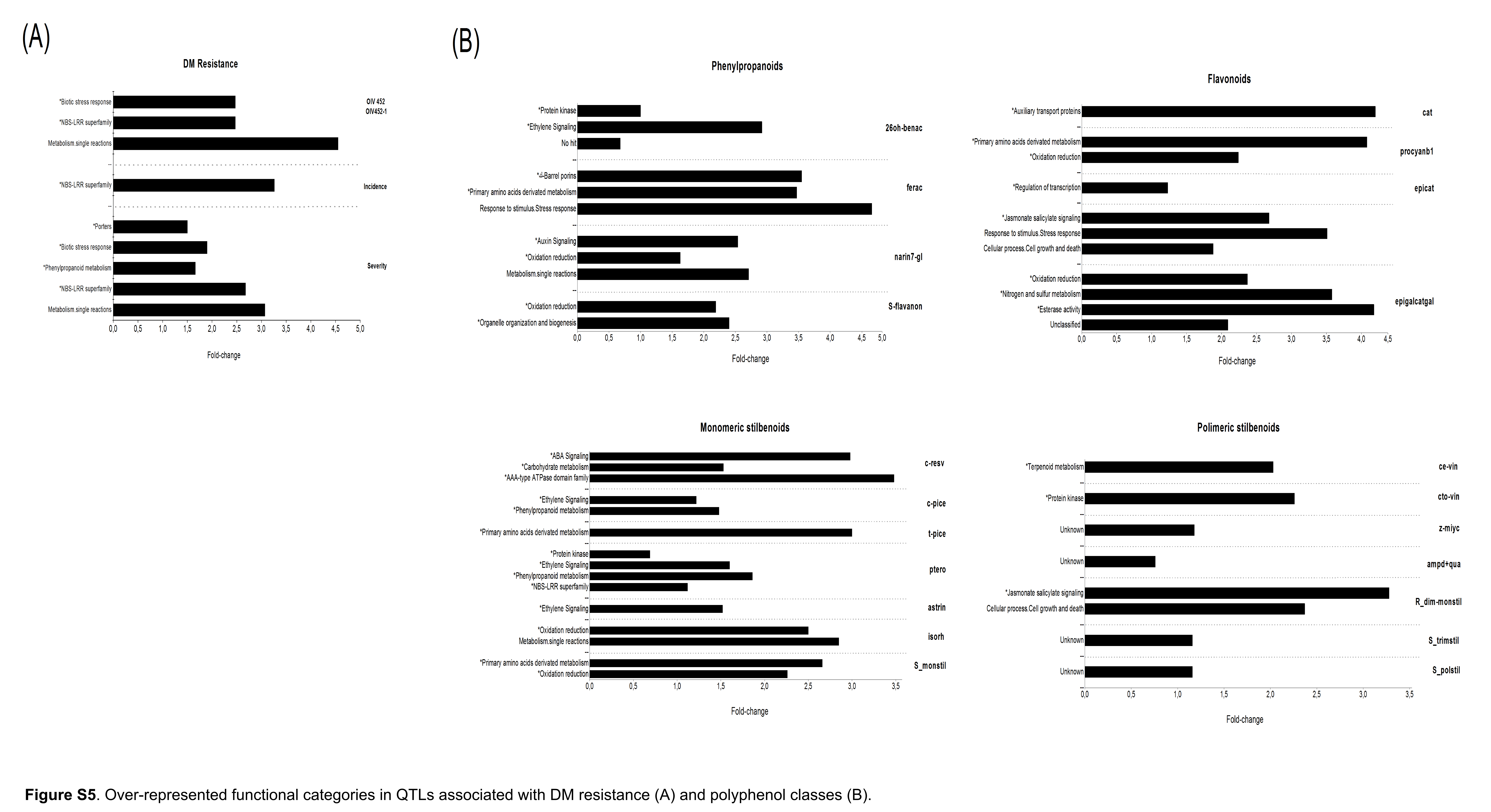

Supplement: Supplementary file 5 [file Image_5.tif]

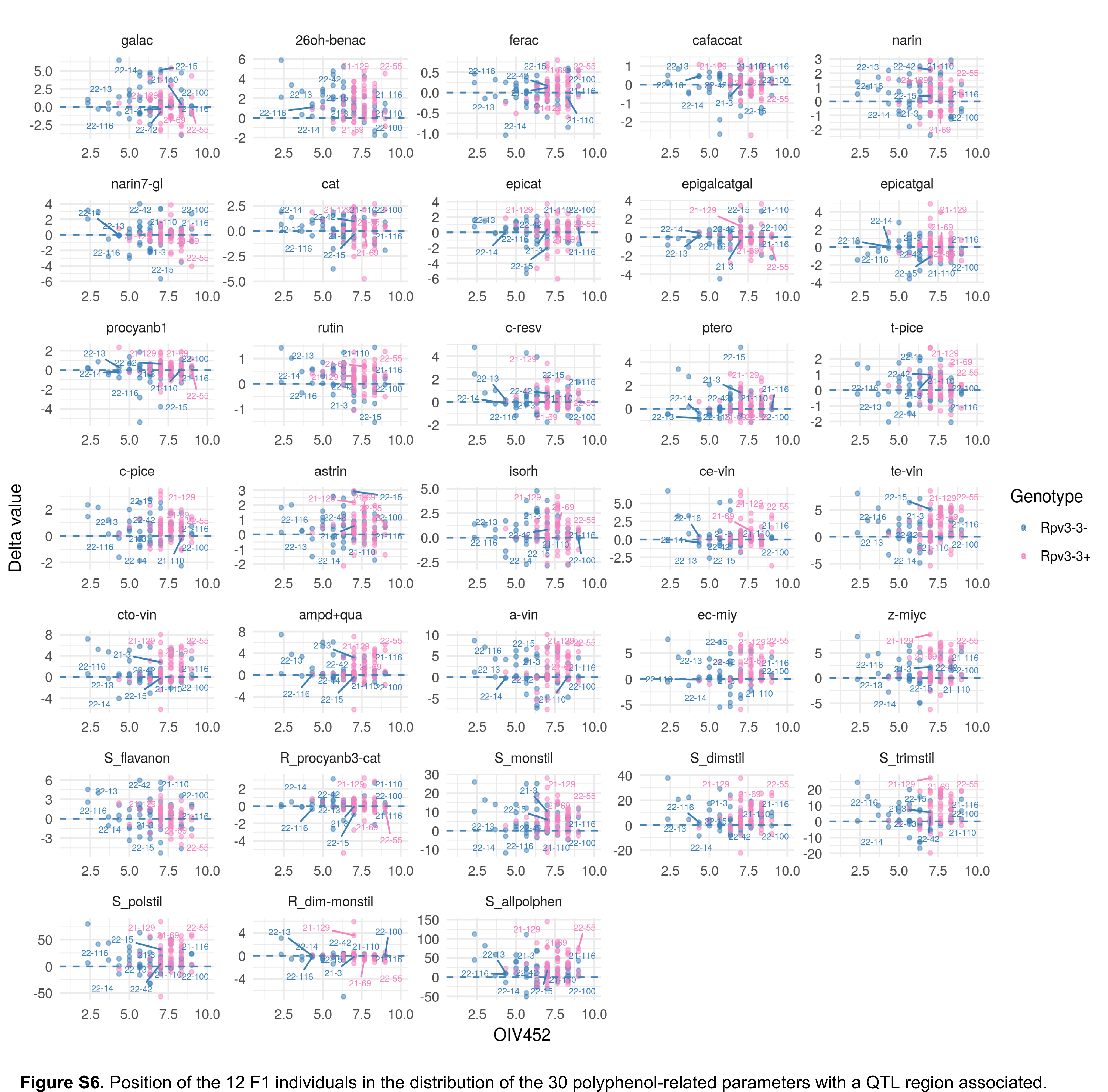

Supplement: Supplementary file 6 [file Image_6.tif]
