# Supplementary material for: The Rpv3-3 Haplotype and Stilbenoid Induction Mediate Downy Mildew Resistance in a Grapevine Interspecific Population
Source: Front Plant Sci. 2019 Mar 6;10:234. doi: 10.3389/fpls.2019.00234 (PMC6414455; doi:10.3389/fpls.2019.00234)

Figure S4. The parental and the consensus M×T linkage map.

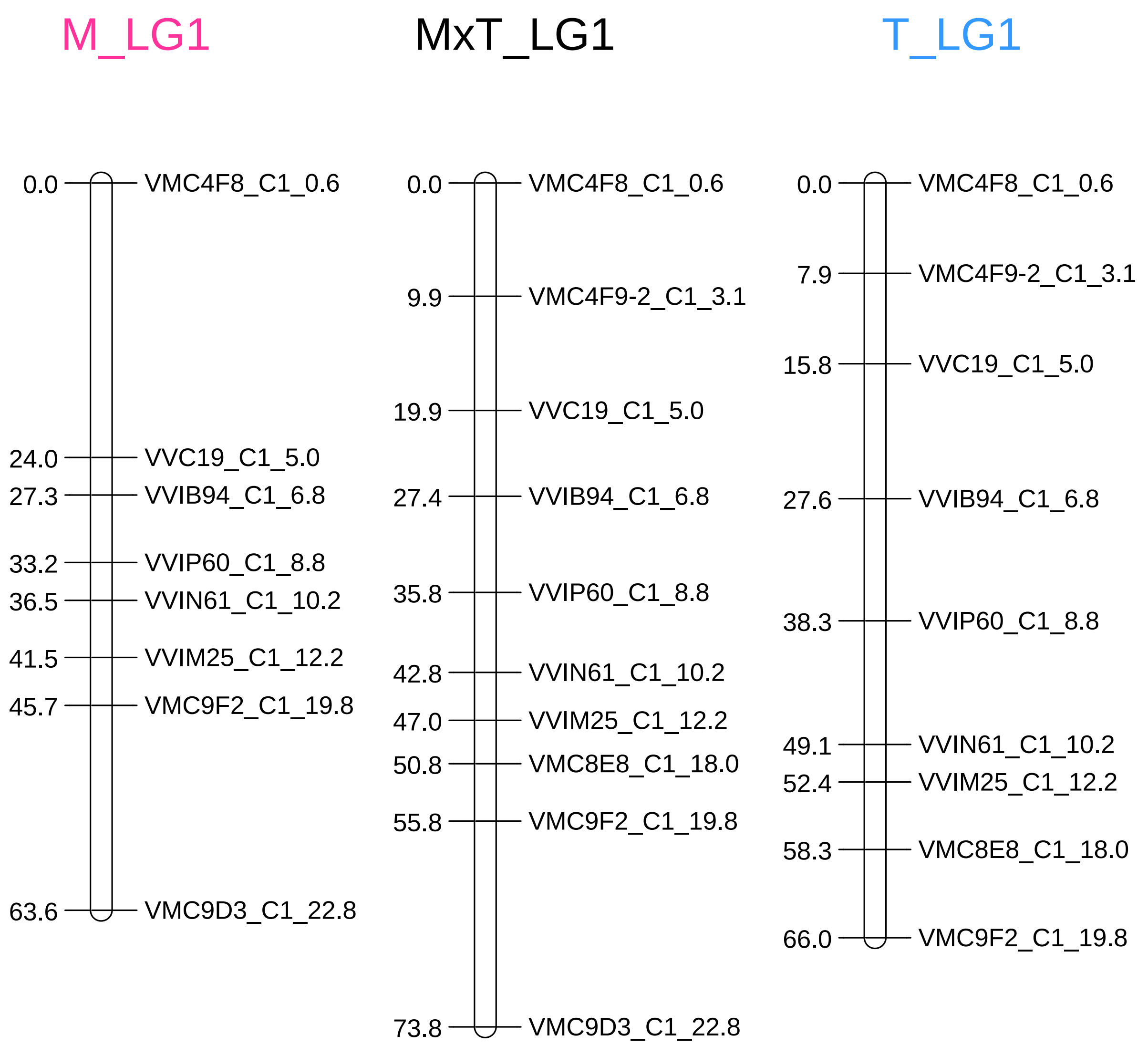

Figure S4. The parental and the consensus M×T linkage map.

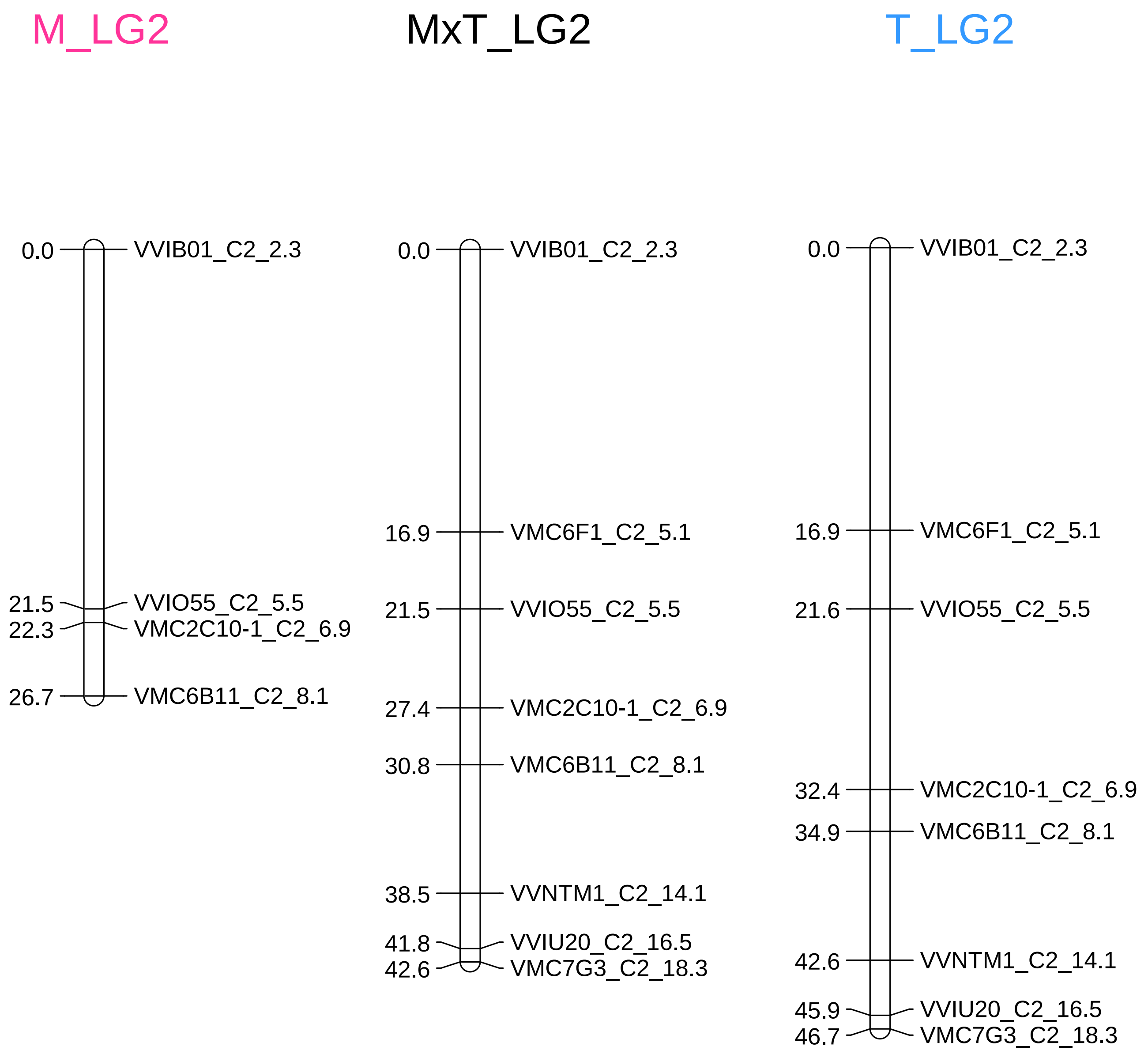

Figure S4. The parental and the consensus M×T linkage map.

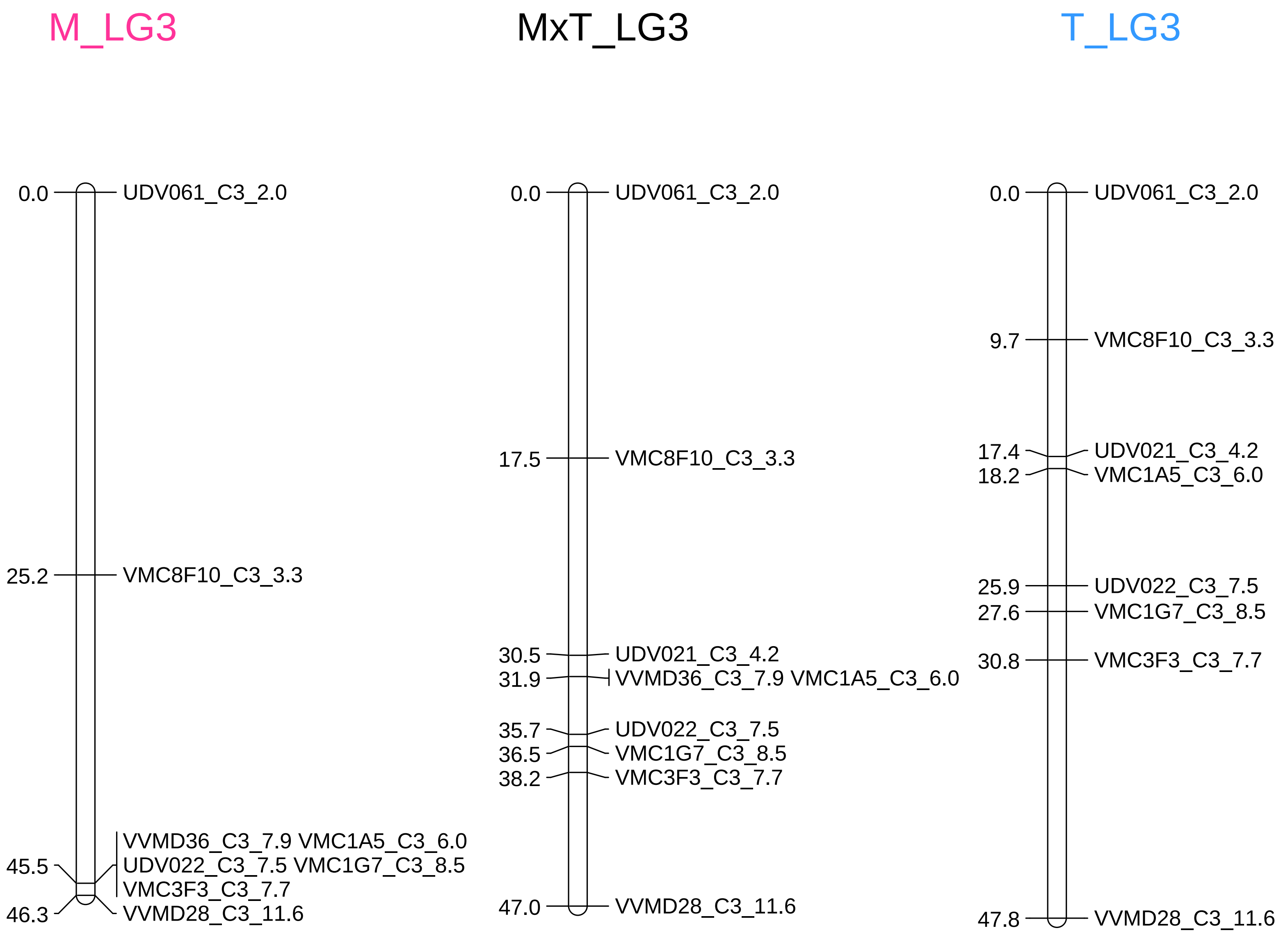

Figure S4. The parental and the consensus M×T linkage map.

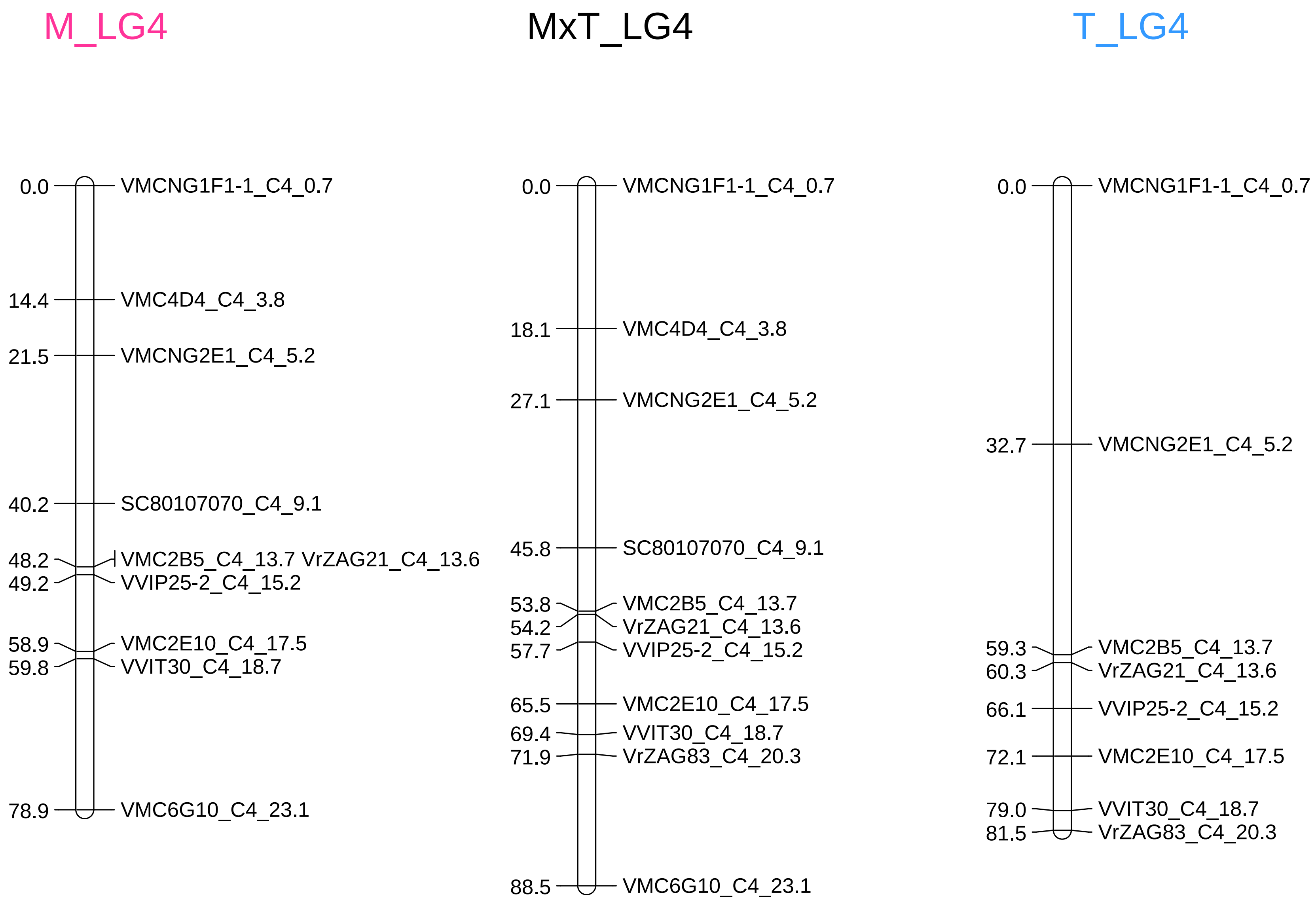

Figure S4. The parental and the consensus M×T linkage map.

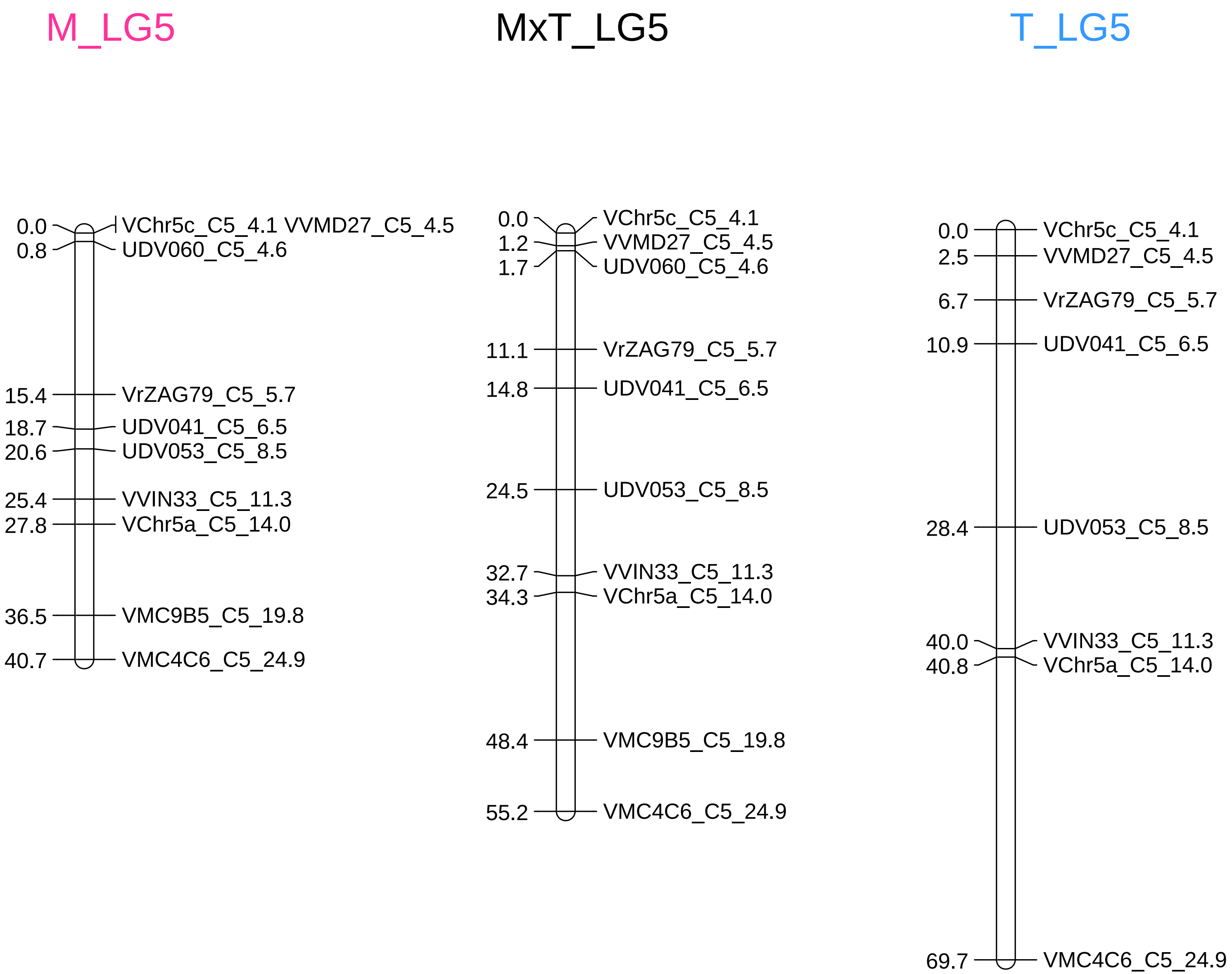

Figure S4. The parental and the consensus M×T linkage map.

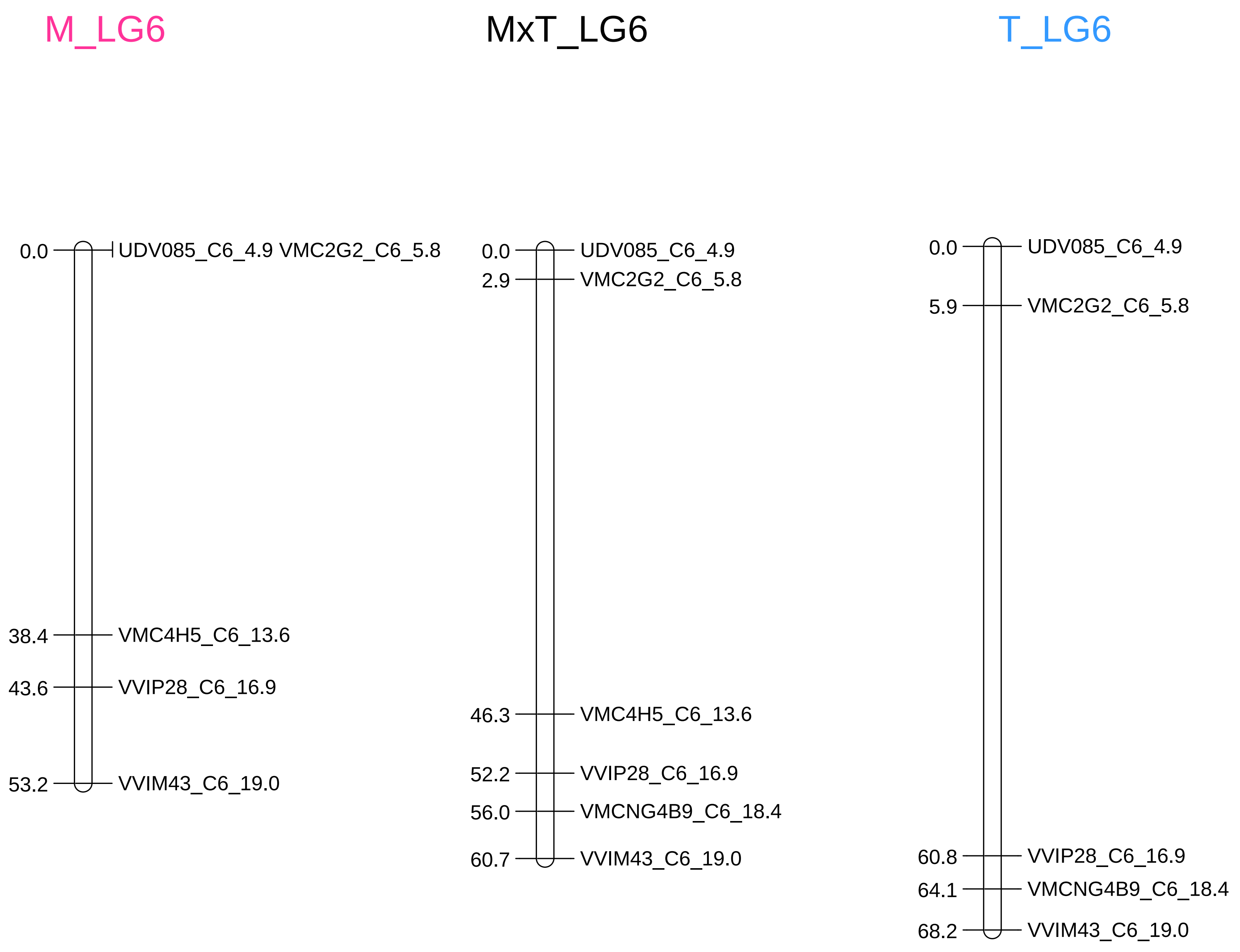

Figure S4. The parental and the consensus M×T linkage map.

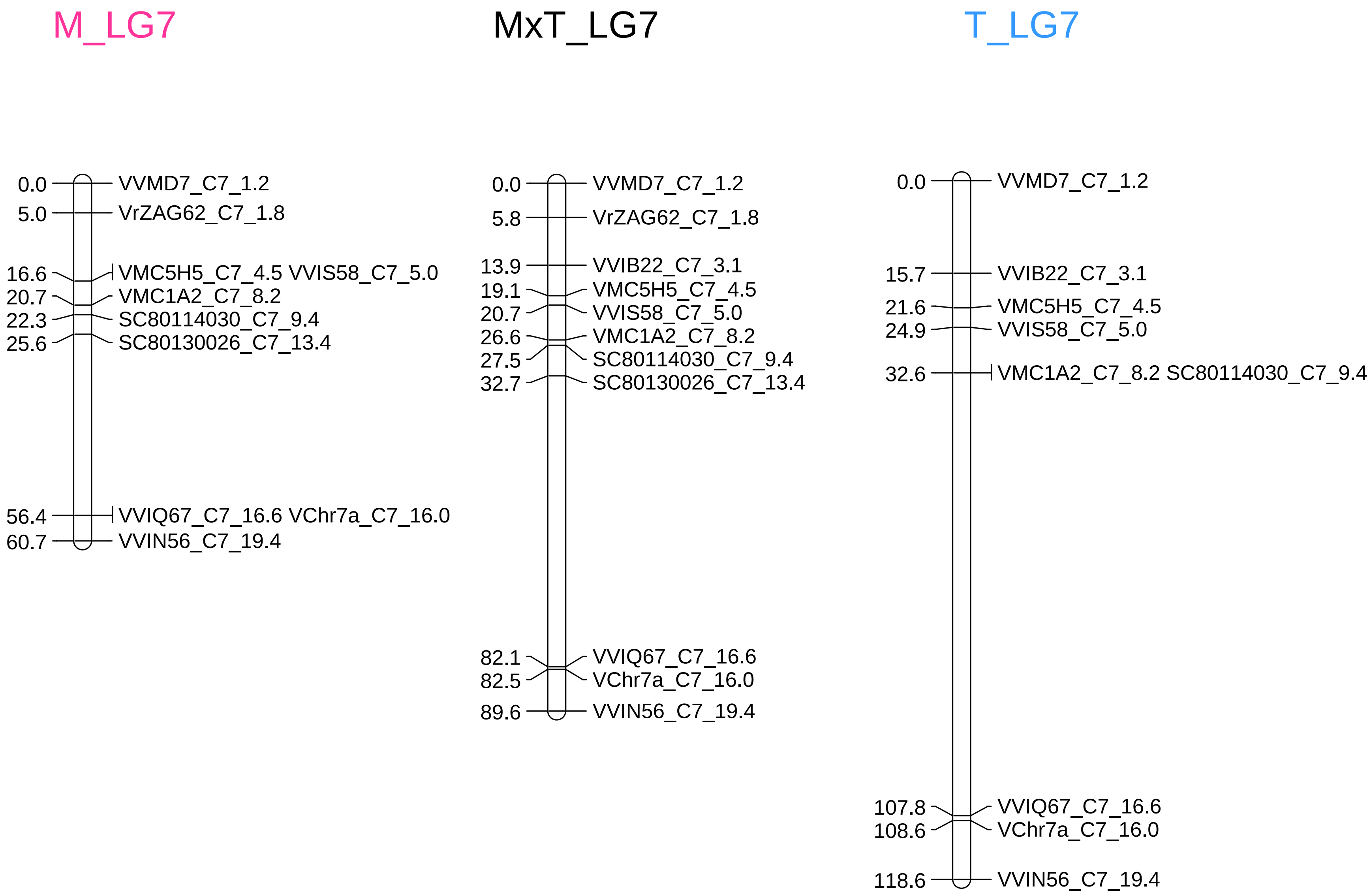

Figure S4. The parental and the consensus M×T linkage map.

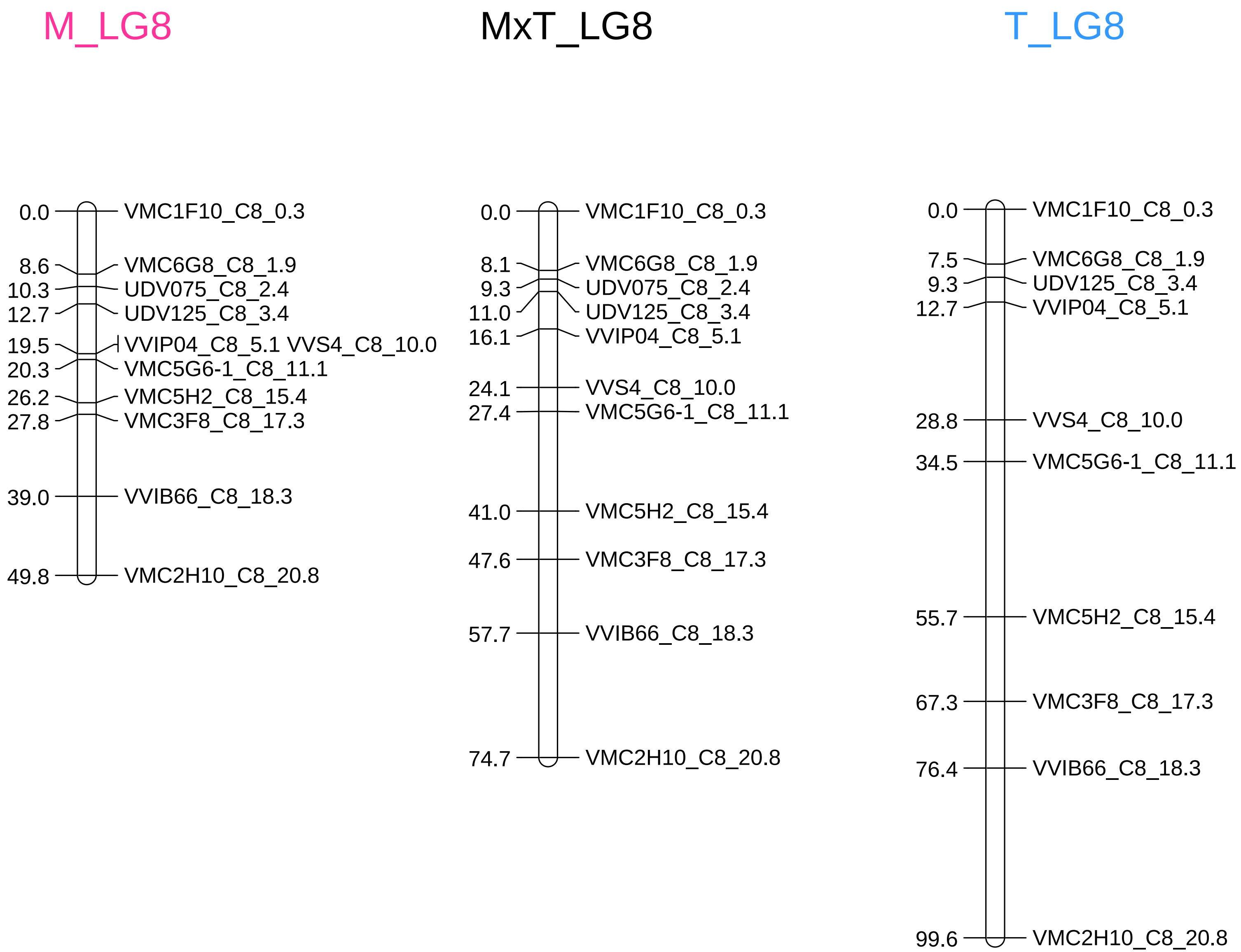

Figure S4. The parental and the consensus M×T linkage map.

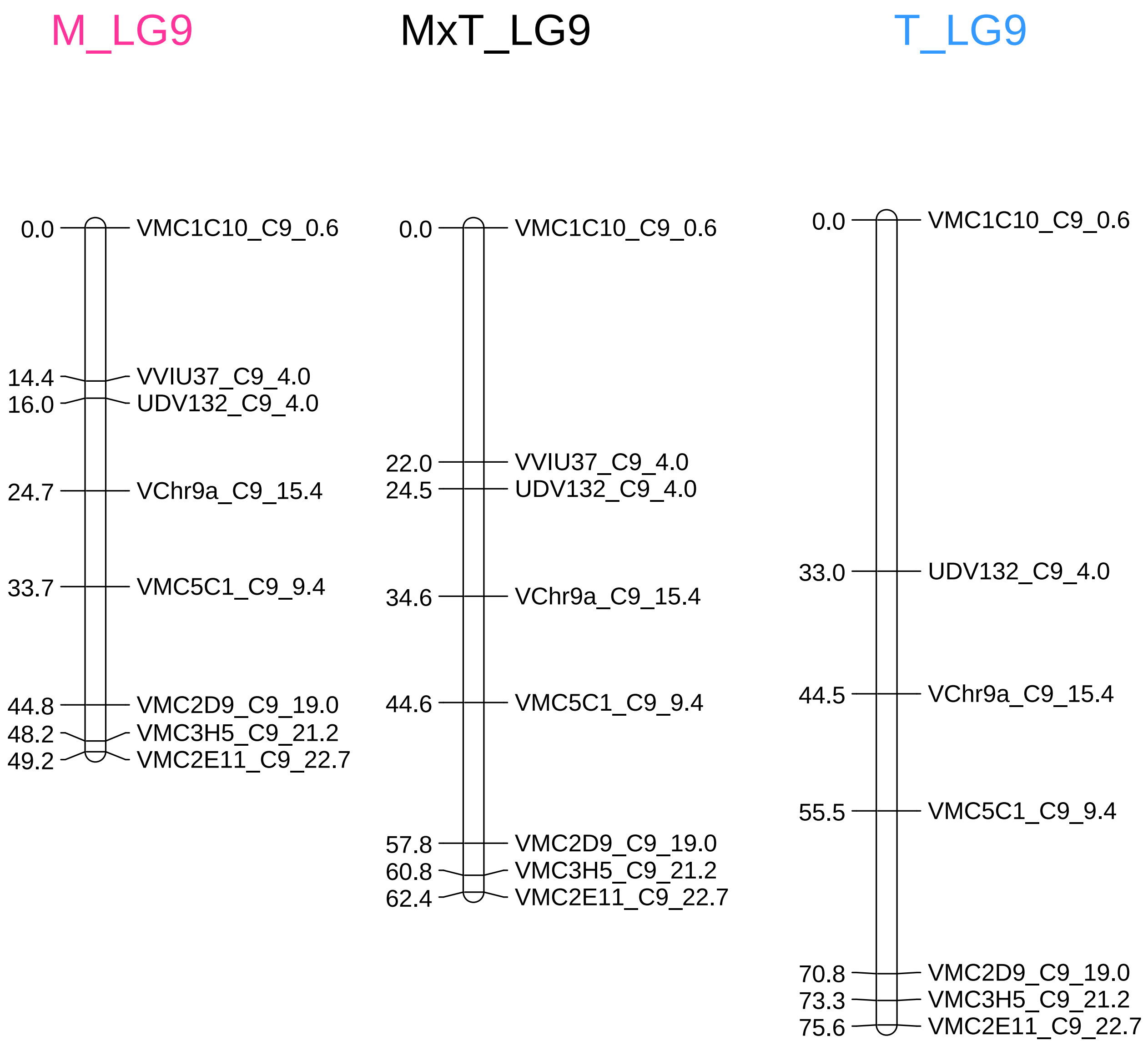

Figure S4. The parental and the consensus M×T linkage map.

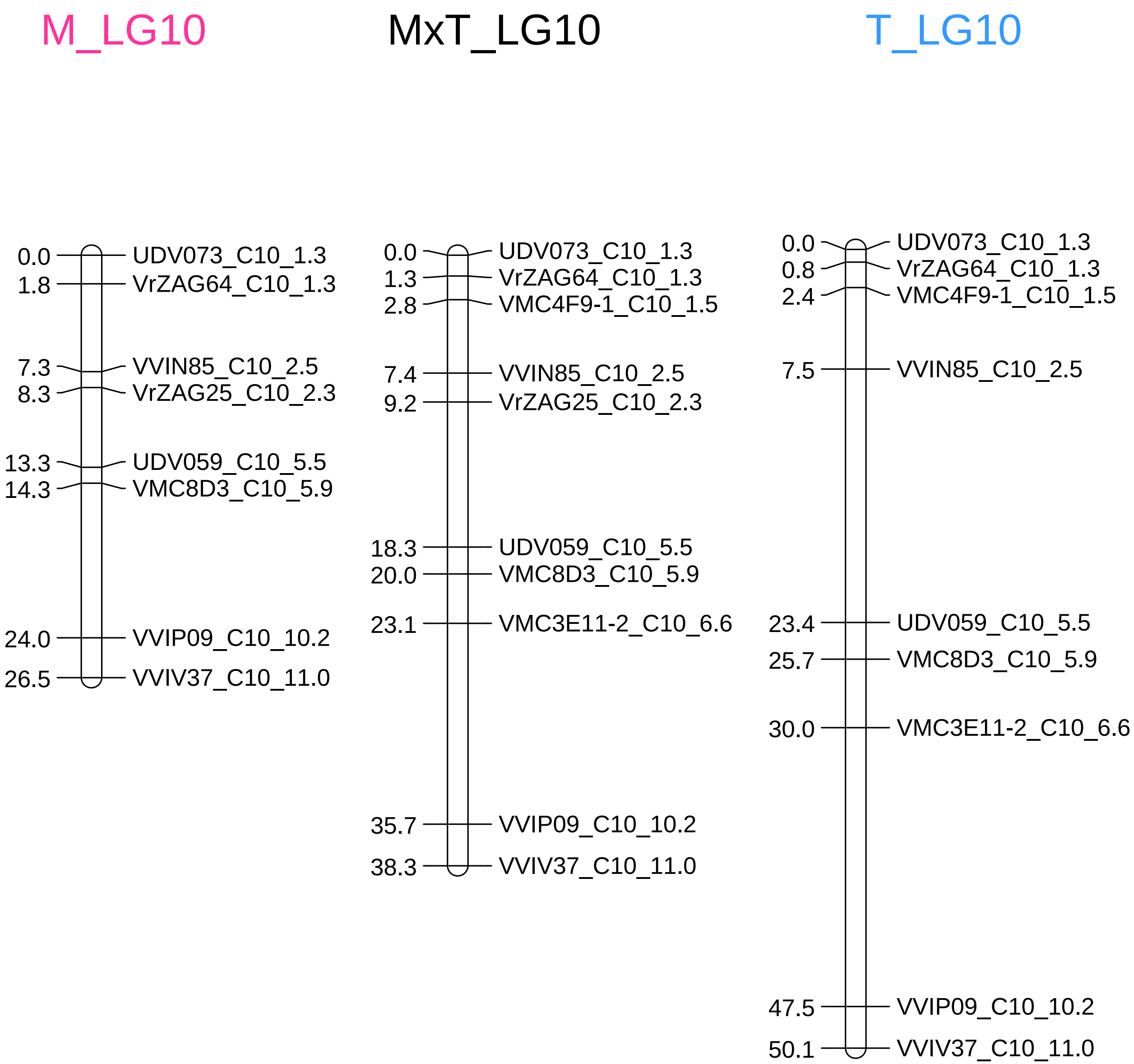

Figure S4. The parental and the consensus M×T linkage map.

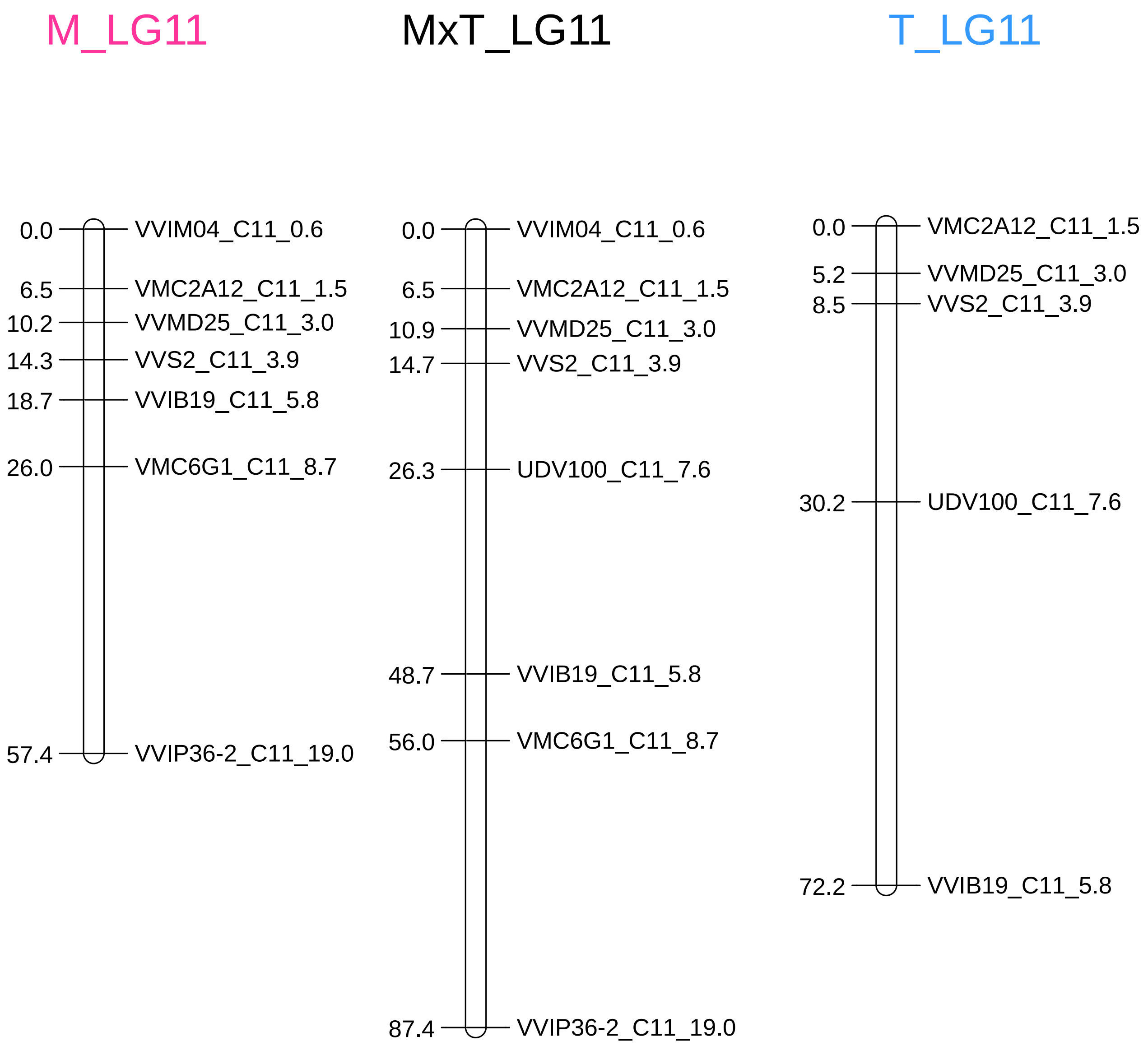

Figure S4. The parental and the consensus M×T linkage map.

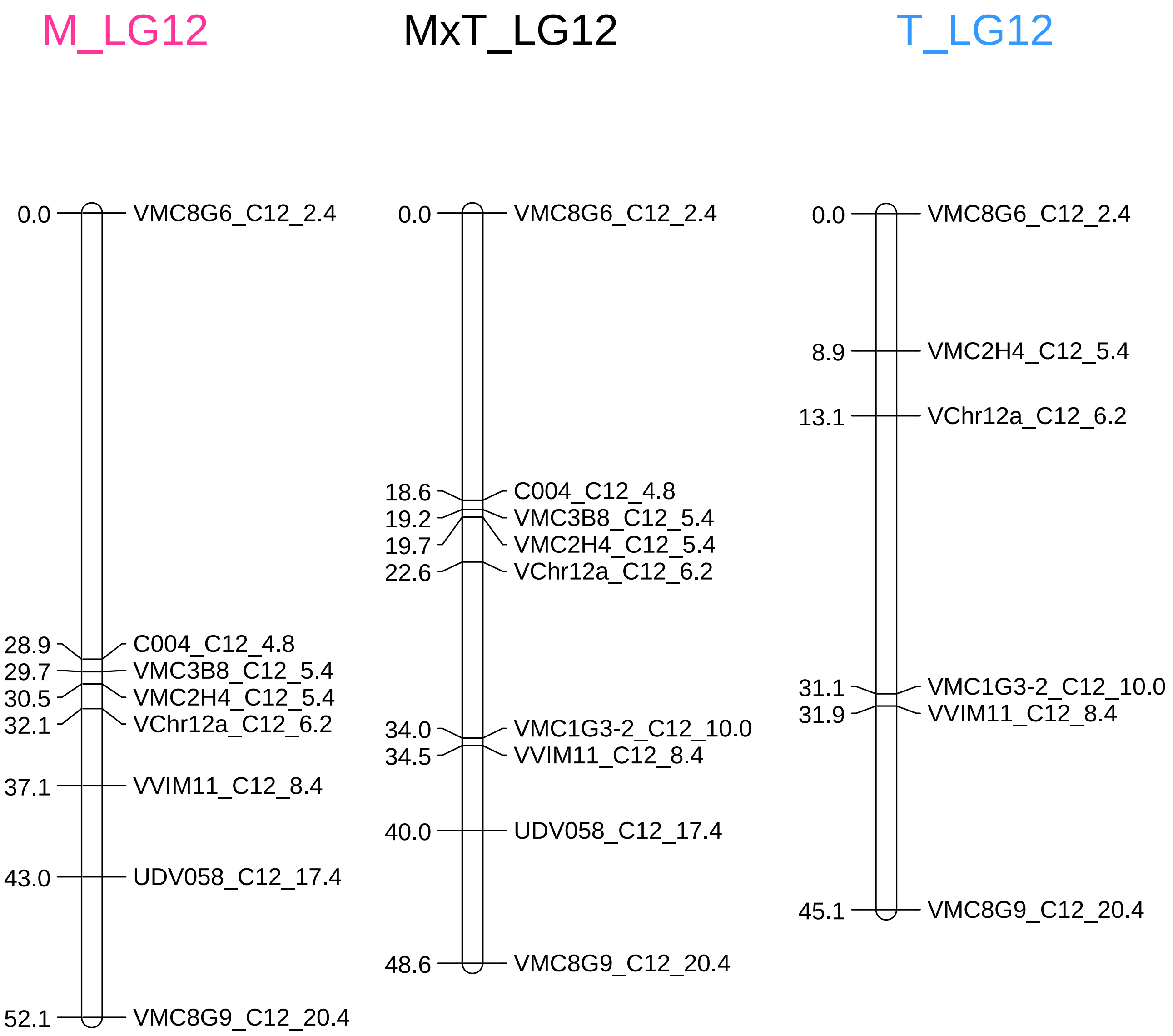

Figure S4. The parental and the consensus M×T linkage map.

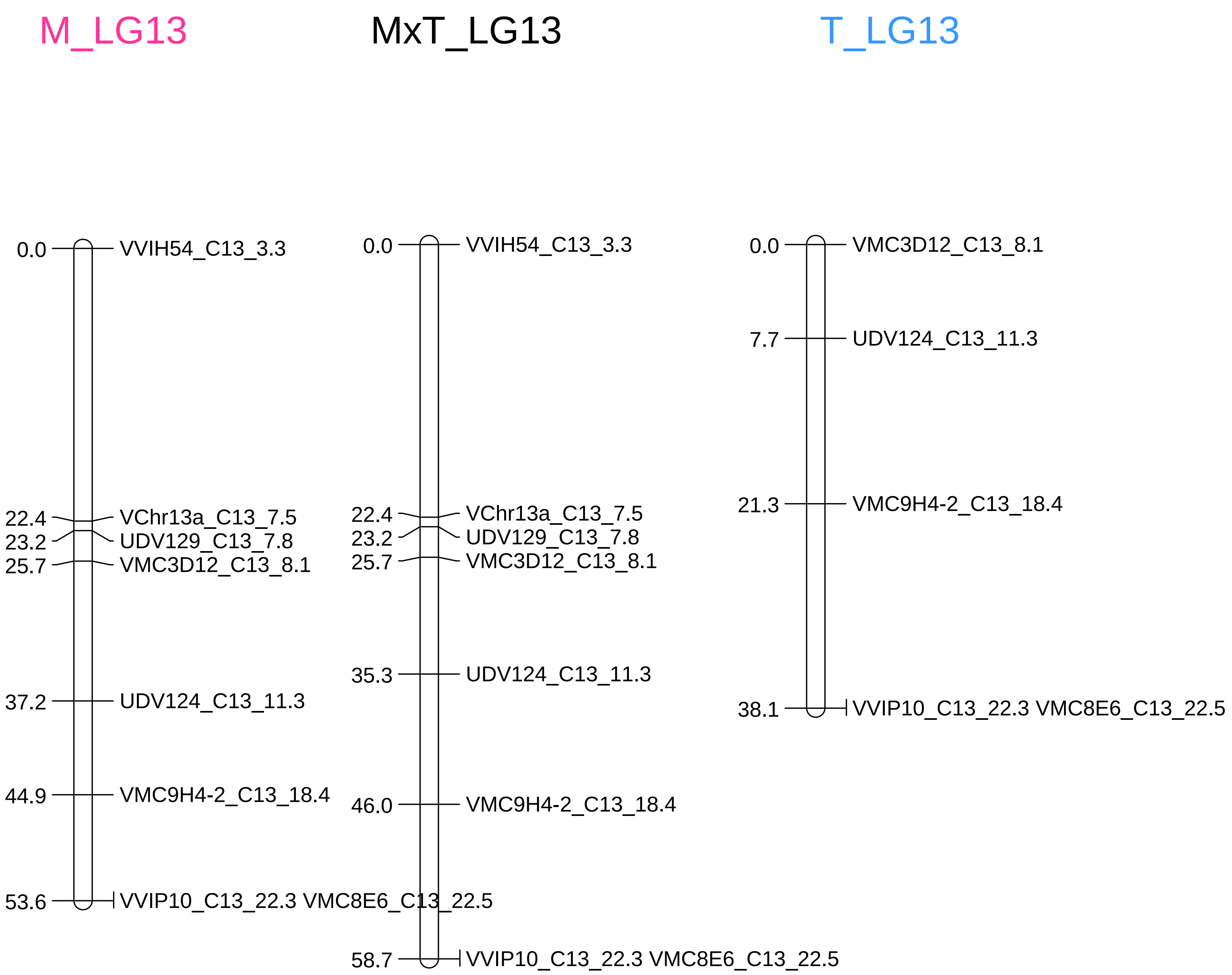

Figure S4. The parental and the consensus M×T linkage map.

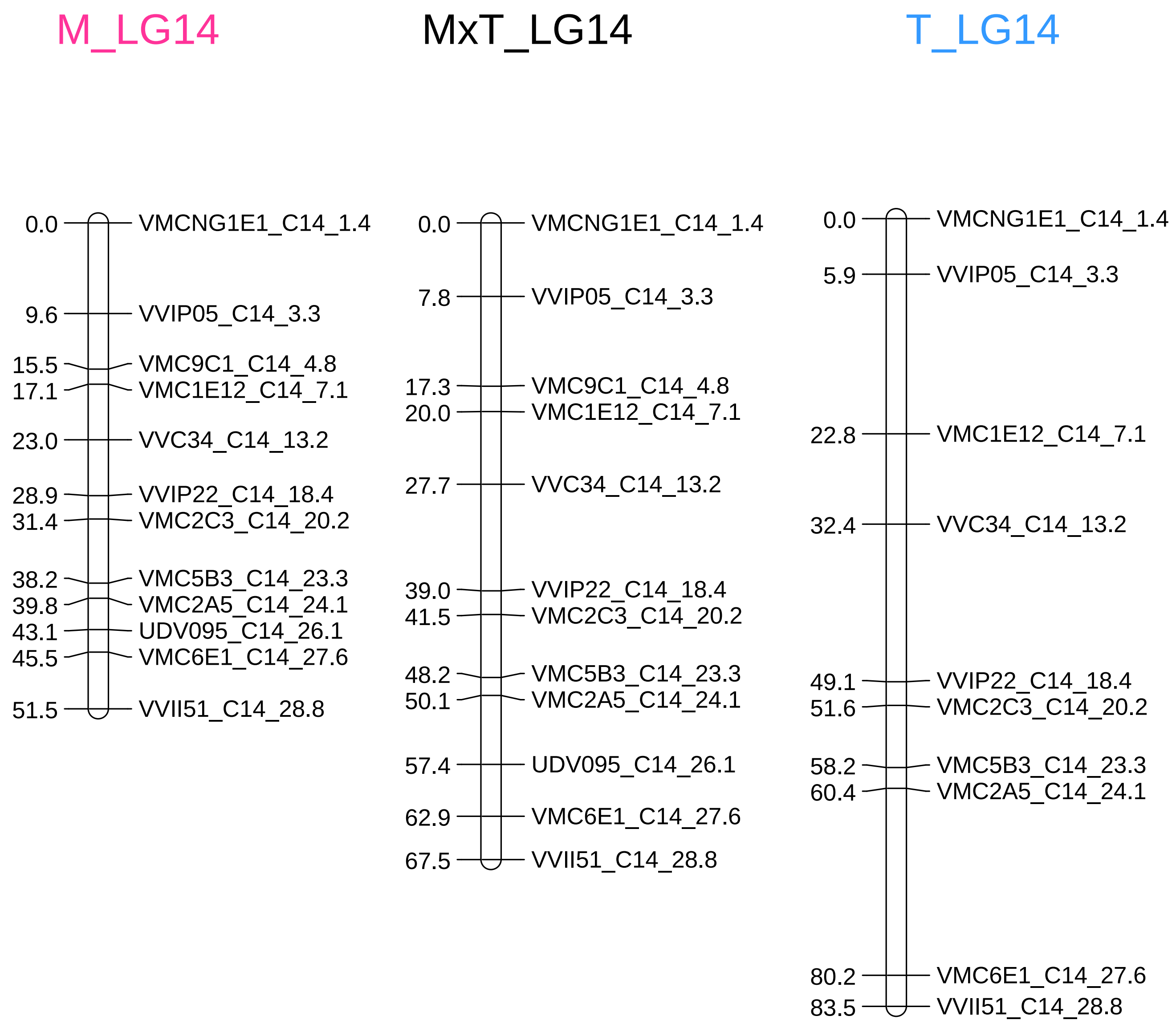

Figure S4. The parental and the consensus M×T linkage map.

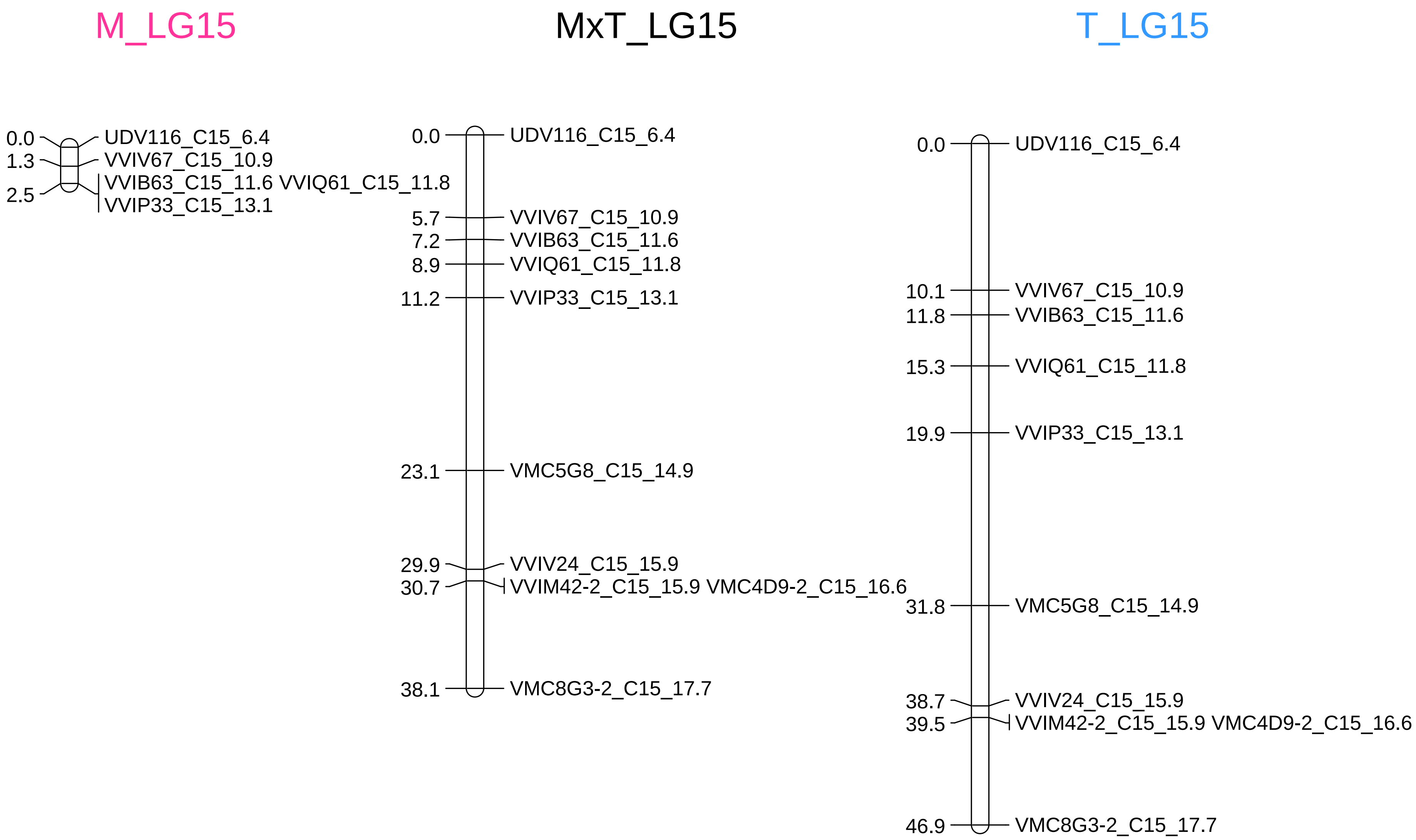

Figure S4. The parental and the consensus M×T linkage map.

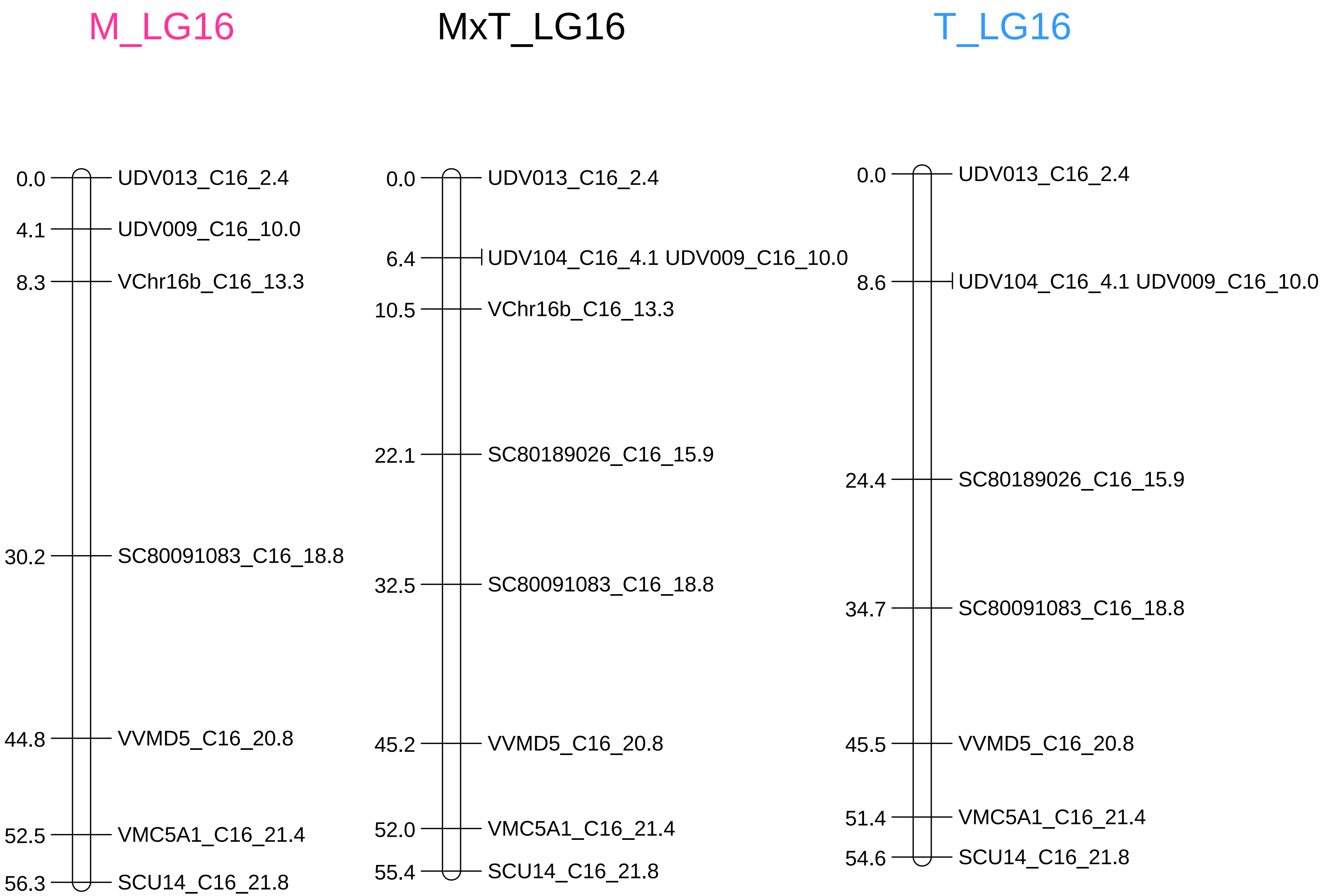

Figure S4. The parental and the consensus M×T linkage map.

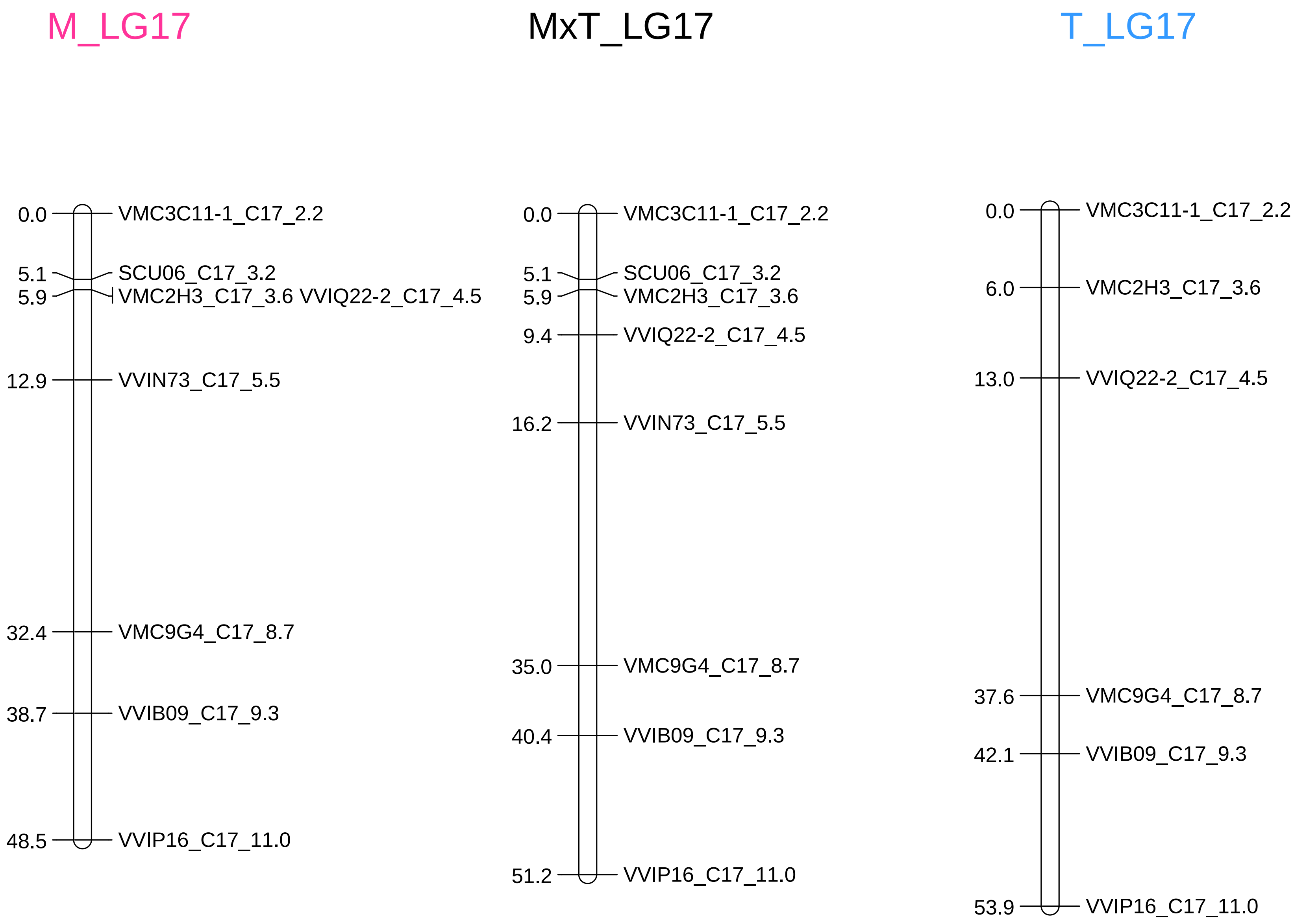

Figure S4. The parental and the consensus M×T linkage map.

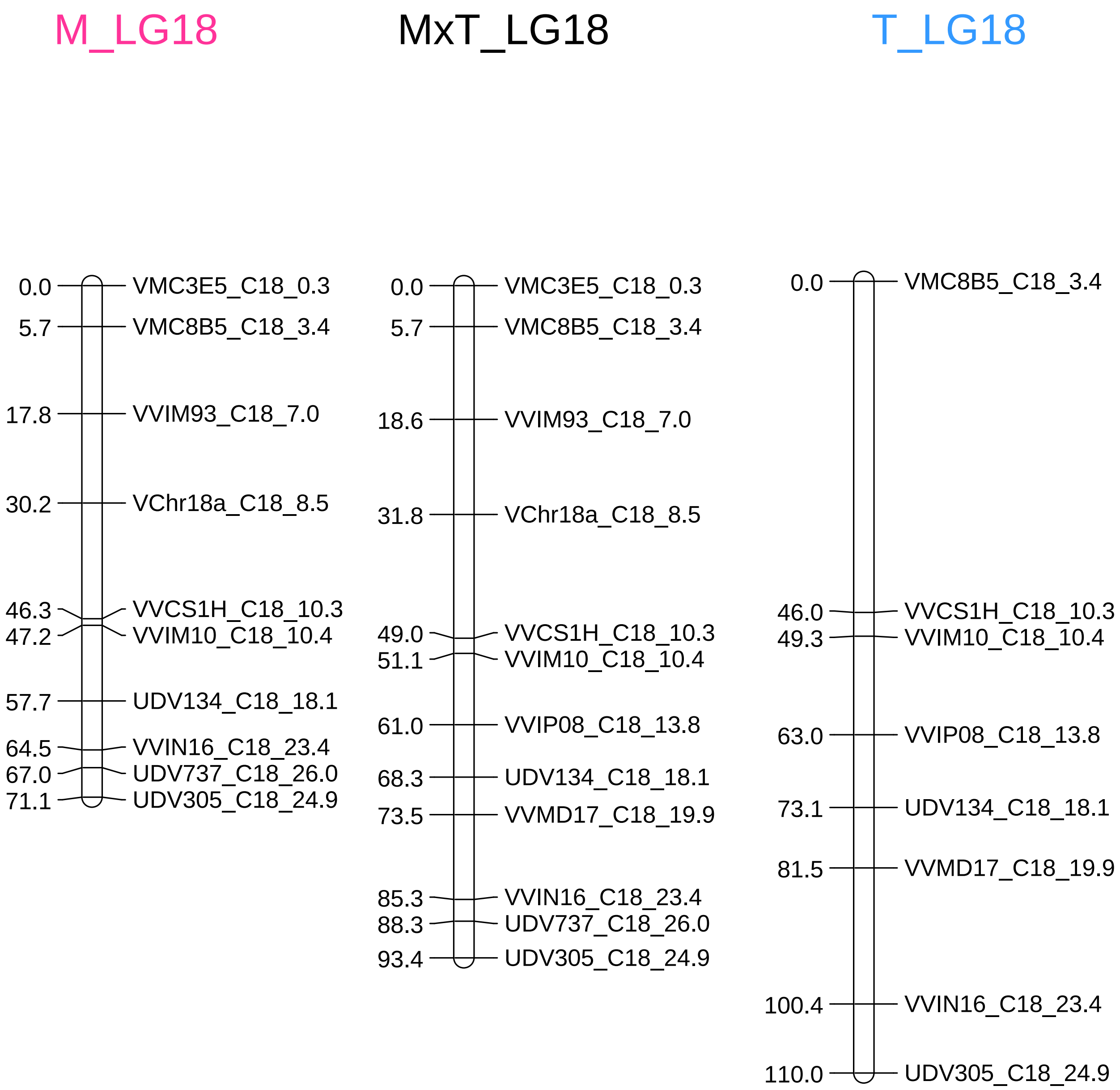

Figure S4. The parental and the consensus M×T linkage map.

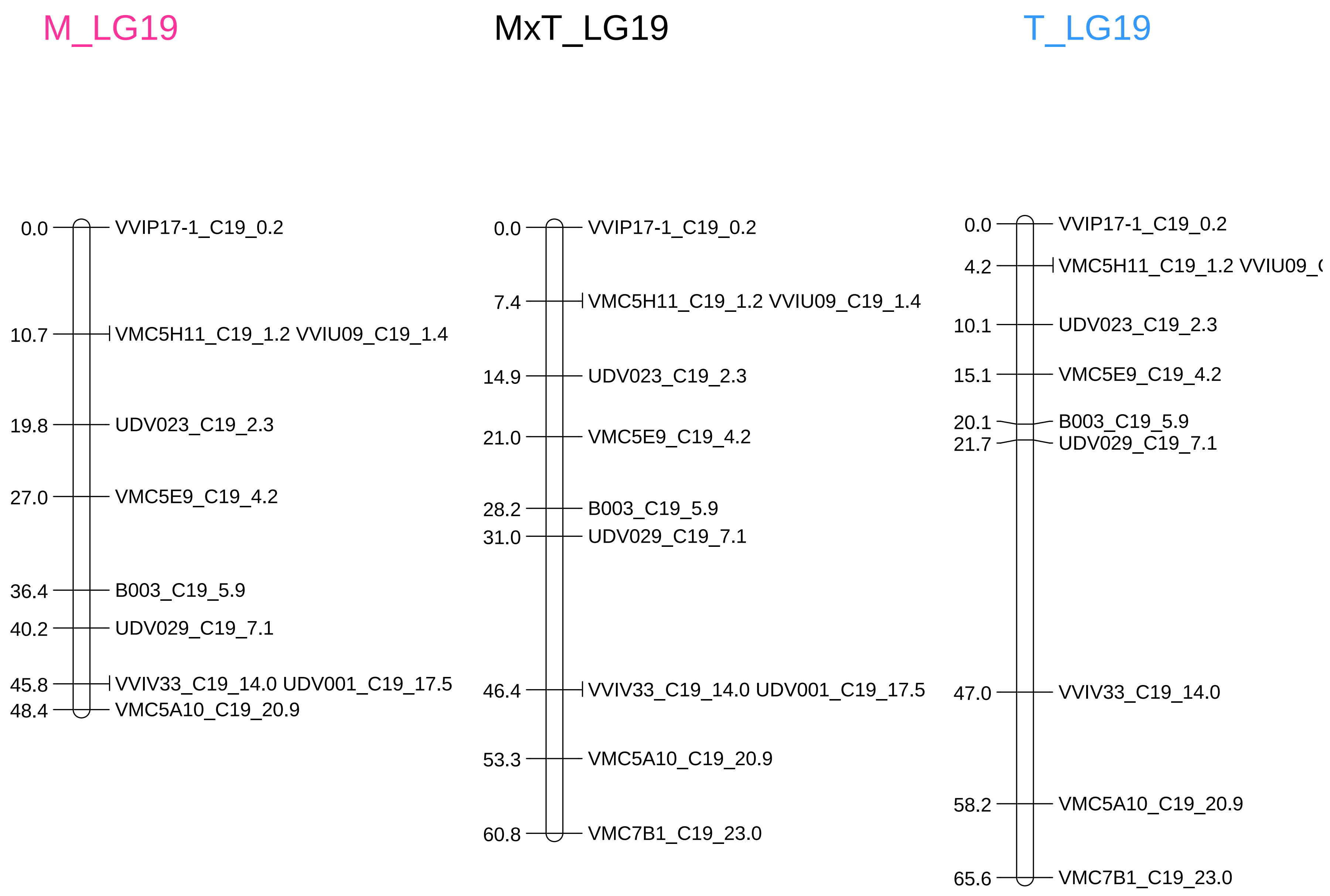

Supplement: Supplementary file 4 [file Image_4.pdf]
